# Active Learning to Understand Infectious Disease Models and Improve Policy Making

**DOI:** 10.1371/journal.pcbi.1003563

**Published:** 2014-04-17

**Authors:** Lander Willem, Sean Stijven, Ekaterina Vladislavleva, Jan Broeckhove, Philippe Beutels, Niel Hens

**Affiliations:** 1 Centre for Health Economics Research & Modeling of Infectious Diseases, Vaccine and Infectious Disease Institute, University of Antwerp, Antwerp, Belgium; 2 Department of Mathematics and Computer Science, University of Antwerp, Antwerp, Belgium; 3 Interuniversitary Institute for Biostatistics and statistical Bioinformatics, Hasselt University, Diepenbeek, Belgium; 4 Department of Information Technology, Gent University–iMinds, Gent, Belgium; 5 Evolved Analytics Europe BVBA, Beerse, Belgium; 6 School of Public Health and Community Medicine, The University of New South Wales, Sydney, Australia; Pennsylvania State University, United States of America

## Abstract

Modeling plays a major role in policy making, especially for infectious disease interventions but such models can be complex and computationally intensive. A more systematic exploration is needed to gain a thorough systems understanding. We present an active learning approach based on machine learning techniques as iterative surrogate modeling and model-guided experimentation to systematically analyze both common and edge manifestations of complex model runs. Symbolic regression is used for nonlinear response surface modeling with automatic feature selection. First, we illustrate our approach using an individual-based model for influenza vaccination. After optimizing the parameter space, we observe an inverse relationship between vaccination coverage and cumulative attack rate reinforced by herd immunity. Second, we demonstrate the use of surrogate modeling techniques on input-response data from a deterministic dynamic model, which was designed to explore the cost-effectiveness of varicella-zoster virus vaccination. We use symbolic regression to handle high dimensionality and correlated inputs and to identify the most influential variables. Provided insight is used to focus research, reduce dimensionality and decrease decision uncertainty. We conclude that active learning is needed to fully understand complex systems behavior. Surrogate models can be readily explored at no computational expense, and can also be used as emulator to improve rapid policy making in various settings.

## Author Summary

Mathematical models are used as pragmatic tools to inform policy makers on public health interventions and many non-health problems. Considerable efforts have been made to build realistic simulation models. Understanding the systems behavior and the effect of model assumptions and parameter values on the results before drawing conclusions for policy is crucial. Common and edge manifestations of complex model runs should be analyzed and therefore we present an active learning approach, also known as a sequential design of experiments, based on surrogate modeling and a model-guided experimentation process. First, we illustrate our approach with an individual-based model for influenza and demonstrate the benefits compared to current complex modeling practices. Second, we establish the power of our approach with a high dimensional model with correlated inputs to explore cost-effectiveness of varicella-zoster vaccination programs. The most influential variables are identified with the aim to reduce dimensionality and decrease decision uncertainty. We also elaborate on the use of surrogate models as an emulator to improve rapid policy making in various settings. To this purpose we provide an interactive platform through which the reader can explore instantaneously the sensitivity of the surrogate models to parameter changes for both our applications.

## Introduction

For many health care interventions, pre-introduction clinical trials are unfeasible for budget or ethical reasons and mathematical models are used as pragmatic tools to inform policy [1]. This is particularly the case for large-scale infectious disease interventions. Simple static fixed risk models are commonly used for health economic evaluation, and sometimes inappropriately so for infectious diseases. Dynamic models representing transmission or evolutionary dynamic systems contributed to our understanding of biological mechanisms and the spread of infections. For instance, in view of its global public health importance, influenza has been the subject of many simulation studies [2]–[10]. The levels of computational complexity and data capacity needs vary substantially between deterministic compartmental models and stochastic individual-based models, the two most widely used types of dynamic models. Such models are developed through an iterative process of designing, coding and validating with empirical data but few have undergone sufficient testing across a range of settings and situations to be fully validated [1]. In order to improve confidence in model-based conclusions, it is necessary to gain a thorough understanding of the system and assess how model assumptions and parameters alter the results and policy decisions [9].

Individual-based models are computationally expensive and can be too complex to fully explore and understand a systems behavior [1]. Different scenarios and parameter values may be explored to account for methodological, structural and parameter uncertainty [3]–[5], [11]. Parameter values can be drawn from a distribution or changed at random over a plausible range. Parameter uncertainty using linear regression in a Latin hypercube design (LHD) is now routinely explored with static health economic models. Unfortunately, these techniques are far less used in the context of dynamic models due to the computational complexity and lack of knowledge on some of the fundamental parameter values and their distributions [12]–[14]. Nonetheless changes in a limited set of parameters or the full set should be explored. Clearly, independent of which method is chosen, it should be transparent and justified in the context of the model [1].

To explore parameter influence, symbolic regression can be used. Symbolic regression enables nonlinear response surface modeling with automatic feature selection. It aims to capture input-response behavior with algebraic expressions without a priori assumptions of model structure [15], [16]. Many variants of this method exist [17]–[19], but here we apply the Pareto-aware symbolic regression (SR) that uses multiple selection objectives [15], [16], [20]. The algebraic expressions are surrogate models for the original computationally intensive simulation model. The model responses can be instantaneously predicted for a set of inputs using the algebraic expressions. These expressions provide information on the relationships between inputs and responses. Ensemble-based SR uses a collection of surrogate models as a final solution and the accordance of the models defines a measure for the prediction uncertainty. Input conditions that are hard to predict indicate that more simulation samples are needed from the corresponding input region.

**Figure 1:**
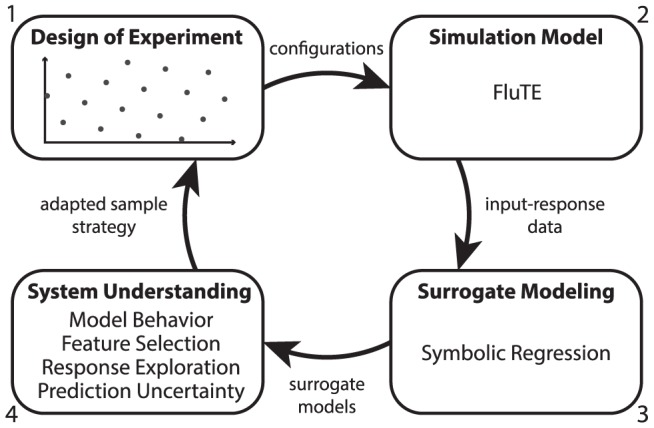
Iterative active learning approach with a simulation model. (1) A Latin hypercube design is used to make configuration files. (2) These configurations are used for the simulation model. (3) All input-response data are modeled with SR. (4) The surrogate models obtained with SR are used to achieve system understanding. The response prediction uncertainty can be used to adapt the experimental design (1) for the following modeling cycle.

The goal of this paper is to present an iterative modeling approach with a model guided experimentation process to systematically analyze both common and edge manifestations of model runs. Figure 1 presents the methodology we recommend to explore simulation models. First, inputs are sampled using a maximin LHD design where the minimum distance between all points is maximized. Second, each point of the input space is used to initialize the simulation model. Next, the input–response data from the simulations are modeled with SR to create surrogate models which can be used for response predictions, feature selection and to identify conditions with large prediction uncertainty. Finally, these insights are used to enhance the experimental design of subsequent simulations by adapting the sampling strategy or reducing dimensionality.

While the general goal is to use a combination of previously described methods from machine learning, known as sequential experimental design [21]–[23] or active learning [24]–[26], we want to emphasize that our approach with SR is an improvement in the field of infectious disease modeling. An iterative modeling approach from [13] was based on step-wise linear regression to estimate response hypersurfaces and was limited to polynomials of the third order. Okais *et al* [14] presented a framework to perform a preliminary sensitivity analysis considering logic and scientific relevance before conducting multivariate sensitivity analysis. However, consistent reproducible methods for the latter were not described. Longini *et al* [2] calculated confidence intervals for disease burden estimates based on prior distributions instead of fixed parameter values, but they did not describe variable importance or system exploration analyses. Van Hoek *et al* [27] performed variable importance analyses without feedback to the experimental design.

We apply our methodology to the open-source model FluTE [8], which is a stochastic individual-based model for pandemic influenza. We illustrate a stepwise system exploration and sensitivity analysis of a computationally expensive simulation model. The model simulates a population with realistic social contact networks and transmission based on the natural history of influenza. Several parameters can be varied to modify the spread of influenza, with the basic reproduction number 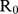 being typically described as the most essential. 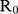 is defined as the expected number of secondary infections caused by a primary infection in a fully susceptible population. For the purpose of our study, we focus on two main outcomes of this model: the clinical attack rate and the day at which the influenza epidemic peaks.

High dimensionality and correlated inputs cause problems in exploring system behavior, which can be resolved through iterative modeling with SR. The approach we present is relevant for many public health problems and we illustrate this through an example of a previously published dynamic model-based economic evaluation of varicella zoster virus (VZV) vaccination [27]. Varicella (chickenpox) is a typical childhood infection caused by VZV and after recovery from chickenpox, the virus may reactivate later in life to cause herpes zoster (shingles). The probability to experience herpes zoster increases with time since the primary VZV infection, but is reduced by natural re-exposure to VZV (e.g., typically parents are re-exposed when their child has chickenpox). Although infant VZV vaccination was shown to dramatically reduce chickenpox morbidity and mortality, there are lingering concerns about its adverse impact on shingles as it reduces VZV re-exposure opportunities [28]. Many modeling and economic studies aimed to tackle this problem (reviewed in [29]–[31]) but the van Hoek *et al* model [32] is of special interest because it includes empirical observations on social mixing patterns and combined childhood and adult vaccination strategies. This age-structured dynamic transmission model was used to perform cost-effectiveness analysis for the United Kingdom [27] with 185 input parameters, including 100 correlated transmission rates, to calculate the incremental gain of Quality Adjusted Life Years (QALY) and costs. Parameter uncertainty was incorporated and conclusions were based on the results of 1000 runs. We analyzed this input-response data with SR to identify driving parameters and compared our findings with a linear regression analysis [33]. Reducing the dimensionality may improve uncertainty analysis since the most influential parameters can be sampled more intensively.

## Methods

The method section follows the approach presented in Figure 1. We provide a guided step-by-step example with basic simulation model as supplementary information (Protocol S1).

### Design of experiments

We used space-filling Latin hypercube designs (LHD) to create parameter sets for the FluTE simulations. In the general case, a sample value from the first interval of the first input parameter is matched at random with sample values from intervals chosen for the other input parameters [13]. Then the second interval of the first input parameter is matched at random with sample values from previously unused intervals of the other features. Each interval of every input parameter will be sampled once and only once. LHD has the advantage that the number of samples is independent of the number of dimensions of the input space but can be determined based on the computational budget, the input dimensions and the complexity of the simulation. Computing a space-filling LHD can be an onerous task and therefore we used the maximin designs of spacefillingdesigns.nl [34], [35]. Because our designs have a rather limited number of sample points, we extended the designs using the Intersite-projected distance method of the Sequential Experimental Design (SED) toolbox [36]–[38].

### Simulation model

#### Influenza model

We made use of an open-source individual-based model for influenza epidemics written in C++, called FluTE [8]. All individuals in the model are members of different social mixing groups. Influenza transmission within each group is based on random mixing. The geographical distribution, employment rates and commuting behavior of the population are based on the 2000 Census data for Seattle (500 000 people) and the Los Angeles County (11 million people), distributed together with the source code of the model. The simulation runs in 12-hour time steps, representing daytime (work, school and community contacts) and nighttime (home and community contacts). Contact probabilities were tuned such that the final age-specific clinical attack rates were similar to past influenza pandemics and observed household attack rates. The model can simulate several intervention strategies based on changes in susceptibility and infectivity due to vaccination or antivirals and on changes in contact probabilities between individuals due to social distancing measures.

#### VZV model

The economic evaluation of VZV vaccination was based on a deterministic dynamic compartmental model [27], [32] with 185 inputs, including 100 correlated transmission rates between 10 age groups. Underlying contact rates were estimated from a survey of social mixing patterns and bootstrapping the original sample specified uncertainty. The model was adapted and calibrated to data from the UK. Source code was not available but we made use of a dataset with 1000 runs, previously subjected to linear regression analysis [33].

### Surrogate modeling with symbolic regression

Symbolic regression (SR) captures input-response behavior by efficiently exploring hundreds of thousands of algebraic expressions of the input variables [15], [16]. Aside from choosing the modeling primitives, no assumptions or restrictions are made on the model structure and genetic programming is used to optimize the search process. SR is a biologically inspired method that imitates Darwin's evolution theory by applying genetic variation and natural selection on the modeling ensemble [39]. We used the SR implementation from the DataModeler package in Mathematica [40]. The result of a SR run is an ensemble of tree-based regression models that give a good approximation of the response variable. The algorithm consists of the following steps [41]:

#### Model initialization

In the first step of the algorithm, a population of SR models is generated randomly and the algebraic expressions of the models are represented by parse trees. Every model is a potential solution that explains the response behavior using the a subset of the input variables. The parse tree of every model consists of terminals and primitive functions. A terminal is either an input variable or a constant. We used a set of primitive functions 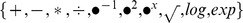 and summation and multiplication have an arbitrary arity. These functions can be adjusted according to the problem domain.

#### Model evaluation

The model fitness is determined by minimizing two objective functions: the prediction error 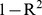, with R being Pearson's correlation coefficient of 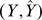 and the model complexity. We define model complexity as the sum of the number of nodes in all possible subtrees of a given tree, which is equivalent to the visitation length, i.e. the total number of links traversed starting from the root node to each of the terminal nodes of the parse tree. The complexity objective is used to avoid excessive growth of the model expressions. Because of the complexity objective, the presence of a variable in a sufficiently evolved population indicates that the variable is relevant for describing the response [20].

#### Model archiving and elitism

After the evaluation of all models, a fixed-size archive of the best achieving models is maintained. This is an elitism strategy that ensures that the best achieving models are never lost after recombination. The archive is populated by a selection of the least-dominated models from both the population and the archive of the previous generation. A model dominates another model if it performs better on at least one objective and does not score worse on any of the other objectives. A model is said to be Pareto optimal if any other model does not dominate it. This way, we can define the Pareto front of a model set.

#### Model evolution

**Figure 2:**
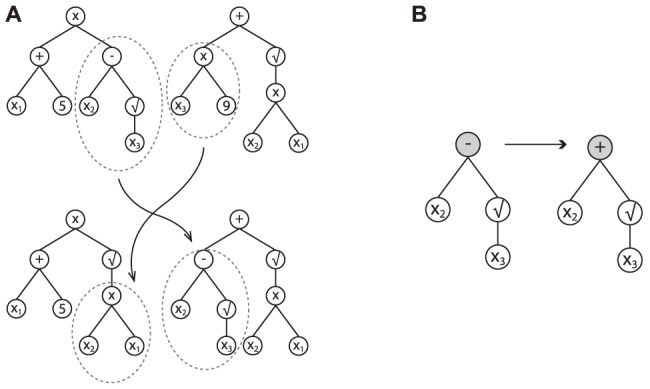
Recombination and modification of algebraic expressions. (A) One point crossover example in symbolic regression. Two individuals swap subtrees, resulting in two new expressions. (B) One point mutation example in symbolic regression. The operator minus is replaced by another operator of the same arity.

A new population of SR models is generated at every step of the algorithm. New models are created with genetic operators like crossover and mutation. Crossover is the process of combining parent models into child models by using subtrees of both parents (Figure 2A). Mutation of a model introduces random alterations in its expression tree (Figure 2B). To select which models will generate offspring a Pareto tournament is held. Models that compete for offspring in the tournament are selected from both the current population and the archive. To generate the offspring, 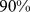 of the children are generated by crossing over two parents obtained from the Pareto tournament. The remaining 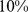 is generated using the mutation operator. Every 10 generations the population is re-initialized with random models to ensure diversity in the population and to counteract inbreeding.

This evolutionary process is repeated over many generations. A maximum number of generations, a time budget or a model accuracy threshold can be used as criteria to stop the process. For this paper we used time budgets based on the size and dimensionality of the data sets. Timings are listed in Table 1 and an example with different time budgets for RUN 3 is described in Text S1.

**Table 1:** Symbolic regression settings.

Name	Value
Population size	1000
Archive size	100
Crossover rate	0.9
Mutation rate	0.1
Population tournaments	5
Primitive functions	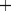 , 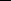 , 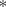 , 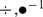 , 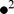 , 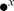 , 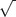 , 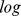 , 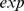
Time budget FluTE RUN 1,2,3	1000 s
Time budget FluTE RUN 4	7200 s
Time budget FluTE RUN 5	3600 s
Time budget QALY	3000 s
Independent evolutions FluTE	8
Independent evolutions QALY	12

### System understanding

Conclusions are based on a model selection from the knee of the Pareto front and we perform nonlinear optimization of the constants within these models. A model ensemble of high-quality and minimal complexity obtained through an effective SR algorithm can facilitate system understanding and focus the research. Variable presence in the final ensemble (taken over several independent SR runs) provides a robust indication of the importance of input variables. Only inputs significantly related to the response can survive a harsh evolutionary pressure and get to the final ensembles [20]. Besides variable importance, final ensembles also provide dimensionality trade-offs in complexity and accuracy of models. Another example of system understanding is the automatically generated hypotheses for meta-variables, low order transformations of driver inputs, which can potentially linearize the final models and enable further application of the powerful linear and regularized linear learning. The sensitivity analysis of constructed ensembles is the highlight of facilitated system understanding. The prediction divergence of the model ensemble is a measure for the prediction uncertainty. Conditions that are hard to predict might be missing from the design.

## Results

### Transmission

We performed a stepwise exploration of the US-tailored simulation model for pandemic influenza (FluTE), applied to Seattle and Los Angeles county [8]. We first simulated epidemics in the Seattle population using four basic model parameters: 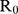, whether individuals can travel, the number of infected individuals seeded into the population and whether this seeding occurs only once (static) or on a daily basis (dynamic). Table 2 summarizes the parameter ranges.

**Table 2:** Parameter design for all modeling iterations with FluTE and obtained variable importance for the cumulative clinical attack rate.

Parameter	RUN 1	RUN 2	RUN 3	RUN 4	RUN 5
Region	Seattle	Seattle	LA County	Seattle	LA County
Travel allowed?	yes/no (−)	yes/no (−)	yes/no (−)	yes	yes/no (−)
R_0_	1.1–2.4 (++)	1.1–2.4 (++)	1.1–2.4 (++)	1.1–2.4 (++)	1.1–2.4 (++)
Infected seeds	0–5000 (+*)	0–1024 (+*)	0–1024 (+*)	0–1024 (+*)	0–1024 (+*)
Seeded daily?	yes/no (++)	yes/no (+)	no	yes/no (−)	no
Ascertainment				0–90% (+)	80%
Ascertainment delay				1–5 d (−)	1 d
Response threshold				0–5% (+)	instant
Response delay				0–30 d (−)	instant
Vaccination coverage				0–90% (−)	0–90% (++)
VE susceptibility				0–66% (−)	0–66% (+)
VE infectiousness				0–66% (−)	0–66% (−)
VE symptoms				0–66% (−)	0–66% (−)
Scenarios	200	200	50	800	200
Repetitions	20	20	10	20	20

++ very important, + important, − almost no impact,

* only small values,

VE: vaccine efficacy.

**Figure 3:**
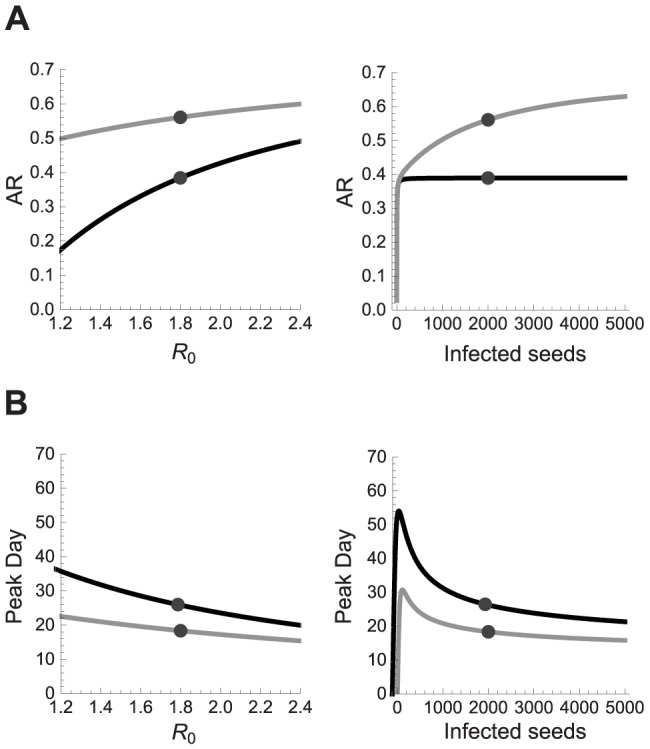
Response prediction plots of the high-quality surrogate models obtained with SR. Response prediction plot for the cumulative clinical attack rate (A) and the day on which the epidemic reaches its peak (B) when seeding occurs only once (black) or on a daily basis (gray). Predictions for 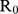 assume a fixed number of infected seeds, indicated by the dot in the panel on the right, and vice versa.

The surrogate models for the AR were of good quality (error <0.001). Although each configuration was executed 20 times, almost no stochastic fadeout was observed. The dichotomous variable indicating whether people can travel was absent in most surrogate models. Given the inherent feature selection of the SR algorithm, this parameter appears to be unimportant to predict the AR [20]. The response plot for the AR (Figure 3A) shows that the number of infected people seeded into the population had almost no impact when seeding once. Only very low numbers of seeded individuals resulted in a different AR. The impact of the seeding number on the AR increased with daily seeding. We observed a correlation of 60% between the AR and the seeding number and frequency though we expected a major role for 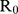.

The day of the epidemic peak advanced logarithmically with an increasing number of infected seeds, although small numbers of seeds could give rise to no or very late peaks (Figure 3B). There is no consensus in the literature on pandemic influenza models about how and to which extent infectious individuals should be seeded. Some studies [4], [8], [42], [43] have been published with static seeding of 1, 10 and 100 individuals while others used dynamic seeding. There seems to be no concern about the potential impact of these different seeding approaches, as only a shift of the epidemic curve due to seeding has been reported [5]. We explored a wide range of seeding values using both static and dynamic approaches, and observed that the seeding approach has impact on the results. The surrogate model divergence for small seeding values was very large so these conditions needed to be sampled more intensively. Model specifications and examples of surrogate models are given in Text S1 and S2.

### Stochasticity

**Figure 4:**
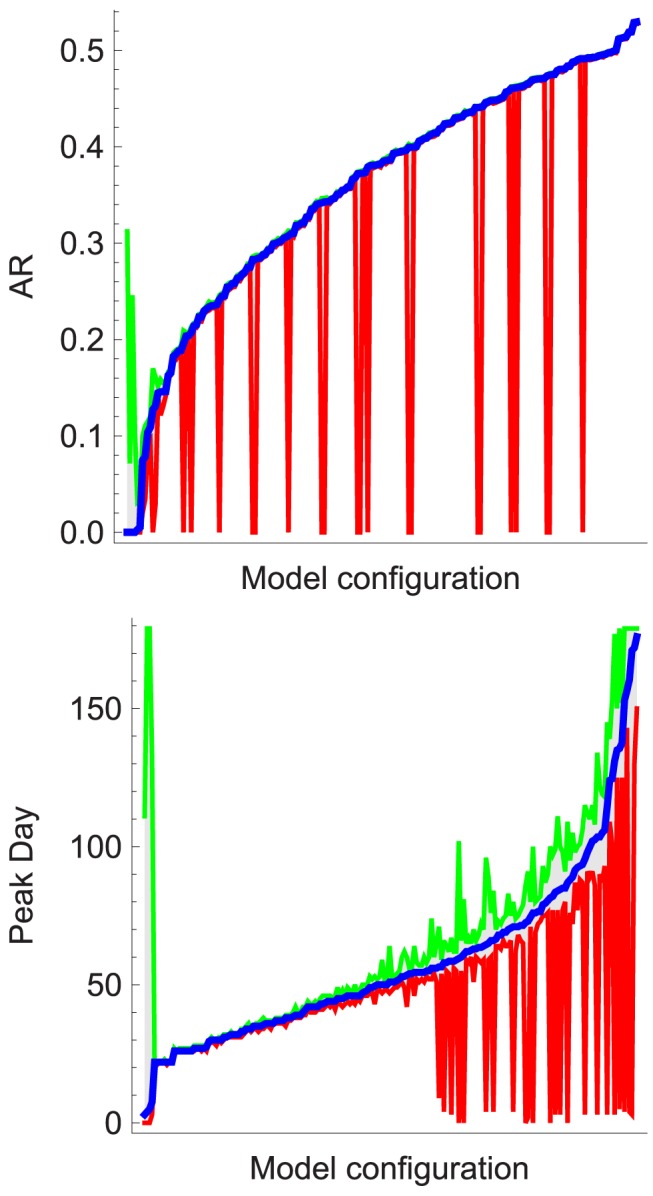
Variability of the simulation model output due to stochastic effects. Multiple executions of the stochastic FluTE model with identical epidemiological configuration lead to different outcomes. The variability is shown by the median, minimum and maximum AR (left) and day of the epidemic peak (right) for each combination of the R0, travel and seeding parameters. The configurations are sorted on their median response value.

With the aim to include more edge cases from the FluTE model, we adapted the initial design (Table 2) by oversampling small numbers of infected seeds with successive powers of two until 1024. This resulted in more stochastic effects, with substantially different output for each parameter set. The current standard is to use average [2], [13], [42], [43] or median [4] results from several realizations of each scenario. Average responses can be very misleading due to stochastic fadeout (AR = 0). Figure 4 presents the median, minimum and maximum AR and epidemic peak day for all configurations. We opted to use all responses for the surrogate modeling to minimize the loss of valuable information. Conditions with stochastic effects increase model divergence. In published pandemic influenza models, the number of repetitions for each scenario ranged from 1 to 1000 [3], [4], [8]. Small numbers have been justified by observing that independent realizations with a given set of parameters lead to very similar epidemic curves [5]. Nevertheless, we observed stochastic fadeout for scenario's resulting in different median AR and especially the day of the epidemic peak seemed sensitive to stochasticity.

### Population

**Figure 5:**
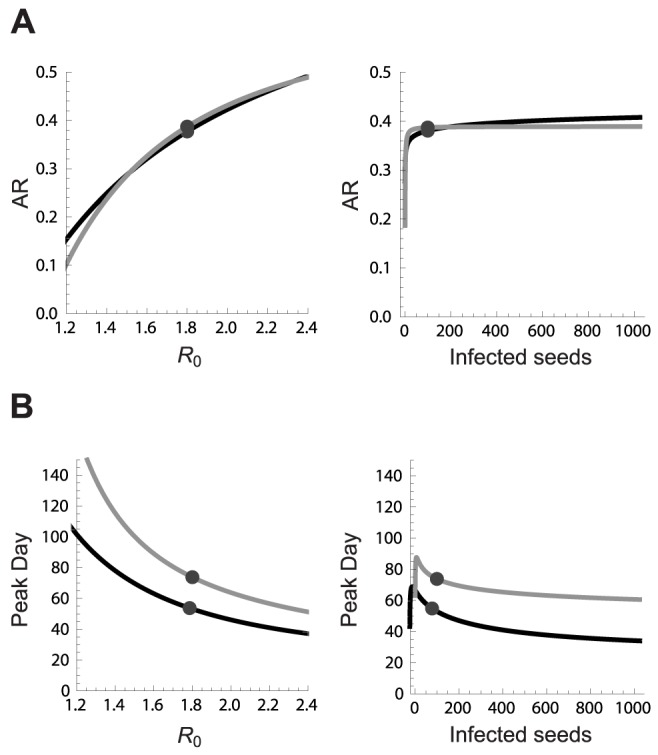
Response prediction plots for Seattle and LA County. Response prediction plots of the high-quality surrogate models obtained with SR for the cumulative clinical attack rate (A) and the day of the epidemic peak (B) in Seattle (black) or LA county (gray). Predictions for 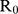 assume a fixed number of infected seeds, indicated by the dot in the panel on the right, and vice versa.

In order to assess the effect of population size, we compared FluTE simulations for Seattle (0.5 million people) and LA County (11 million people). We used a single design with four transmission parameters for both populations (Table 2) and compared the surrogate models of each dataset. We observed similar response predictions for the AR (Figure 5A), indicating that this outcome is insensitive to population size, when population size is already substantial (i.e. 0.5 m). The travel parameter was absent in most surrogate models for both populations, indicating that this is inherent to the simulation model. The main difference for the enlarged population was the timing of the epidemic (Figure 5B). For example, a pandemic with 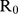 = 1.8 and 100 infected seeds would result in an AR of 0.38 for both populations, but the epidemic peak day in LA County is predicted to be 15 days later compared to Seattle. The similar AR and postponed peak for the larger population are in line with results of previous studies [8], [44]. We did not compare urban and rural regions due to lack of data although this may have a large impact [4]. Model ensemble divergence for low seeding numbers was less for LA County, which suggests that large populations absorb stochastic effects.

### Vaccination

After adjusting the transmission settings, seven parameters for reactive vaccination strategies were added to the design (Table 2). The computational burden to simulate Seattle was much lower compared to the LA County. Therefore, we used the Seattle population for the initial exploration with vaccination parameters. Based on the resulting input-response data, surrogate modeling showed that mainly the response threshold and ascertainment fraction were important to predict the AR. The importance of 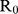 and the vaccination coverage increased when the response threshold and ascertainment parameters were set to mimic instant reactive measures, immediately after emergence.

### Emulation

**Figure 6:**
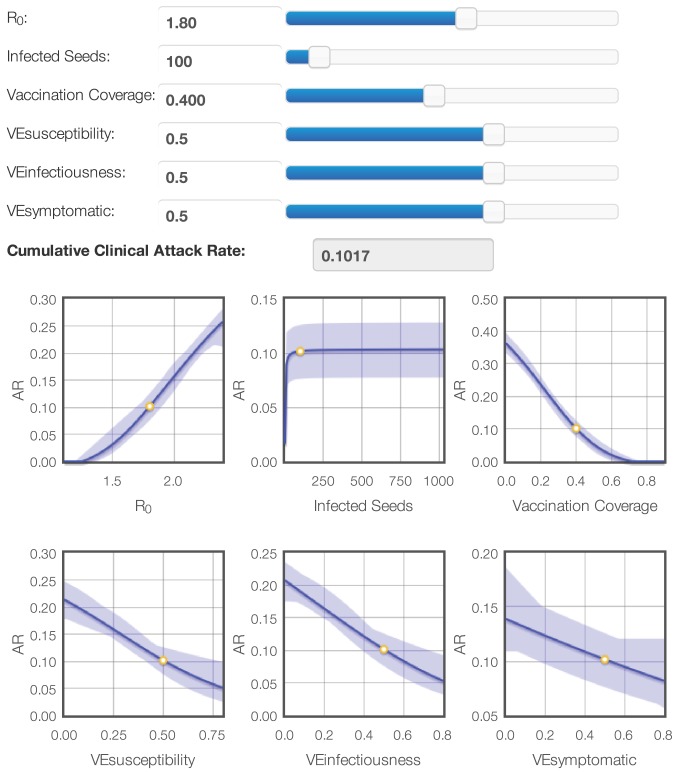
Response plot explorer for the cumulative clinical attack rate. An interactive version of this plot is available at www.idm.uantwerpen.be (see Text S4).

After subsequent simulation and modeling iterations, we obtained surrogate models for LA County that can be used to explore reactive vaccination policies on the outcome of ongoing pandemics. Figure 6 shows a basic interface to visualize the response behavior by changing the surrogate model parameters. When vaccination coverage is set to zero, the results from the second design emerge again (Figure 3). Further exploration of the surrogate models revealed a saturation effect of the vaccination coverage on the AR. The predicted AR with a vaccination coverage of 60% is almost zero for 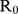 = 1.8 and vaccine efficacies of 0.5. The protection of the general population by vaccination of a subset is known as herd immunity [45]. The clear visualization of herd immunity with the surrogate models emphasizes the usefulness of our approach since it is hard to observe this effect directly from the numerous individual simulation results. An interactive version of this plot is available at www.idm.uantwerpen.be (more info in Text S4).

### Feature selection

**Figure 7:**
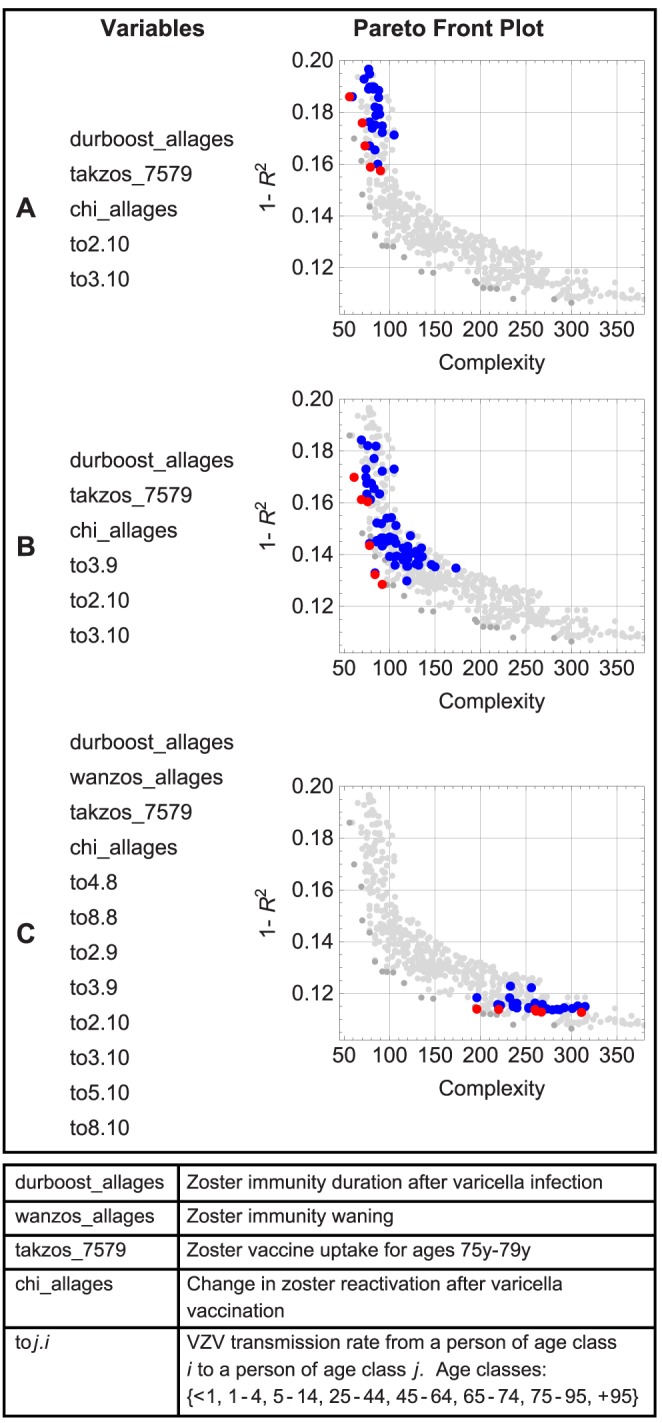
Model error (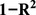) and complexity of high-quality models for the incremental QALY. Models with the variable combinations presented on the left are shown in color (red = Pareto front). Only the most abundant combinations are given: (A) Surrogate models with five variables, (B) Surrogate models with extra variable (to3.9) and decreased model error compared to (A), (C) Surrogate models consisting of twelve variables with small model error but high model complexity.

Timely and effective identification and easy exploration of important variables enhances our understanding of the underlying system. SR can be used for feature selection and is capable to handle high dimensionality and correlated variables [20]. Here, we focus on the surrogate modeling step of our approach with a dynamic transmission model to explore the cost-effectiveness of infant and adult VZV vaccination options. We analyzed the results from an economic evaluation described in van Hoek *et al* [27] with 185 inputs, 100 of which are correlated transmission rates. Parameter uncertainty was explored by using 1000 different configurations. One of the essential findings based on this model [27], [46] is that the incremental Quality Adjusted Life Years (QALYs) might become negative, suggesting infant VZV vaccination might in some cases do more harm than good. Hence the pivotal issue for policy making is to identify and explore the variables that determine the incremental QALYs. Therefore, we performed a SR analysis with all 185 variables to model the vaccination benefits, expressed as incremental QALYs. The model error and complexity of the surrogate models with the most abundant variable combinations are presented in Figure 7. We obtained surrogate models with only five variables with a model error of 16% (panel A), which indicates strong correlation between these inputs and the response. With the sixth variable in panel B, a small reduction in model error can be achieved. The panel C models show further decreases in model error, but also substantially greater complexity due to the presence of additional variables. Some of these variables are important to predict the response, but others may mostly increase complexity without explaining the response. Especially with high dimensionality and model complexity, it is possible that some less important variables remain captured in the surrogate models. To tackle this problem, we performed a second SR analysis with the twelve variables from panel C. This way, we reduced the number of drivers for the incremental QALYs to eight and ended up with surrogate models with an error of only 10%, which is an improvement compared to the models from panel B. Surrogate model specifications and examples are listed in Text S3 and a response plot interface is presented in Text S4.

Marginal contributions were estimated with SR by inspecting the decrease in model error by adding variables. We observed a correlation of 47% between the incremental QALY and combinations of the zoster immunity duration and the change in reactivation due to vaccination. The zoster vaccine uptake for ages 75–79 y was responsible for another 11% increase in correlation. Using linear regression, Bilcke *et al* [33] obtained similar results. They estimated the marginal contribution of all transmission rates at 29%, but they were unable to select particular age-specific transmission rates due to their strong interdependency. Using SR on the same data set, we found that this 29% contribution was explained by just three of the 100 transmission rates.

High variable importance came from rates of transmission from adults to children, despite the fact that mainly children would be in the infectious state pre-vaccination. The transmission rates are based on symmetric contact rates, implying only age-specific differences in susceptibility and infectiousness account for this observation. To study the age component of the transmission rates we estimated the incremental QALYs once with the southwest corner of the transmission matrix and once with the northeast corner. Transmission rates between similar age groups were selected in both experiments but the models with the adult-to-child transmission had a slightly lower model error. It is not exactly clear why this is the case. These variables may capture adult-child interactions, which are relevant for re-exposure to VZV, as well as the susceptibility of children in the presence of universal vaccination. However, this does not imply that transmission would occur more often from adults to children than the other way around.

## Discussion

We present an iterative process of active learning with SR for the systematic exploration of simulation models. Our initial experimental setup for the pandemic influenza model showed only a subset of the systems behavior but provided insights leading to an improved design. We explored common and edge manifestations and ended up with an ensemble of surrogate models for the complex simulation model. The surrogate models for reactive immunization strategies revealed effects like herd immunity and can be useful to instantly evaluate reactive strategies for specific 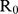 values based on plausible estimates for vaccination coverage and efficacy.

Although we demonstrated these methods on two vaccination programs, based on two distinctly different dynamic models (stochastic individual-based and deterministic compartmental), we are certain that these methods are relevant to address a wide range of public health problems that are informed by modeling. Surrogate modeling with SR identified the most important variables. A decrease in the uncertainty of these parameters would improve the robustness of the simulation results. Also, the feature selection can be useful during the development of the simulator. E.g., the travel parameter does not seem important in the current FluTE implementation although other studies have stressed the role of travel restrictions on epidemics [5], [6], [44], [47]. A revision of the travel implementation may be required.

Considerable efforts have been made to build realistic simulation models of high quality, but most of these are not fully explored. Ideally, each model should be analyzed systematically to understand system behavior and to assess the impact of model assumptions and parameters on the results. The availability of simple surrogate models based on complex simulation models not only serves to understand the original complex model better, but also to emulate it. Policy makers can easily use an interactive interface, such as the ones we present in this paper, to mimic the context in which their decisions take place (e.g., transfer model outcomes between broadly comparable countries) and predict the effectiveness or cost-effectiveness of health interventions. In that sense, the use of surrogate models as emulators provides a great opportunity to enhance both the understanding of these models and improve the reliability and speed of policy making based on existing elaborate model structures. Specifically, for FluTE we could instantly formulate some insights from the emulator (Figure 6) in clear language for policy makers. First, we predict that without reactive measures 36% of the population will be infected. Second, only a few imported cases are enough to start the epidemic hence (complete) isolation may delay (prevent) the epidemic. Third, 30% vaccination coverage (percentage of the population vaccinated) may result in a 55% reduction in the number of cases and 60% coverage in a 95% reduction due to indirect protection because of the interruption of transmission pathways in a partial immune population.

In future, we aim to automate the iterative surrogate modeling approach in order to speed up the process and make it more accessible. The number of realizations should be analyzed more into detail. We acquired already substantial insights on the transmission and vaccination dynamics implemented in the FluTE model with five iterations. However this system exploration could be further expanded, for instance by considering other interventions (e.g. social distancing) separately or in combination with vaccination. While the presented epidemiological results are acquired using the previous generation of simulators, we argue that our approach is applicable to all simulators and should be used for testing and validation when new simulators are developed, and for the emulation to aid policy making across settings after that.

## Supporting Information

Protocol S1 Step-by-step example of active learning.
(ZIP)

Text S1 Symbolic regression analysis FluTE.
(PDF)

Text S2 Optimized high-quality model ensemble RUN 1.
(PDF)

Text S3 Symbolic regression analysis QALY.
(PDF)

Text S4 Response plot explorer.
(PDF)
