# Supplementary material for: Active Learning to Understand Infectious Disease Models and Improve Policy Making
Source: PLoS Comput Biol. 2014 Apr 17;10(4):e1003563. doi: 10.1371/journal.pcbi.1003563 (PMC3990517; doi:10.1371/journal.pcbi.1003563)
Supplement: Protocol S1 — Step-by-step example of active learning. (ZIP) [file pcbi.1003563.s001.zip › StepByStepExample.pdf]

Protocol S1: Step-By-Step Example of Active Learning

Problem domain

In this example, we use the ACKLEY function as core for our simulation model.
[D. H. Ackley. “A connectionist machine for genetic hillclimbing”. Boston: Kluwer Academic Publishers, 1987.]

$$f\,(x, a, b, c, d) = -a * e^{-b\sqrt{\frac{1}{d} * \sum_{i=1}^{d} (x_i\hat{}2)}} - e^{\frac{1}{d}\sum_{i=1}^{d} (\mathrm{Cos}[c\, x_i])} + a * e$$

```
f[x_,a_,b_,c_,d_]:=-a*Exp[-b*Sqrt[(1/d)*Sum[(x[[i]]^2), {i,1,d}]]]
        -Exp[(1/d)*Sum[(Cos[c*x[[i]]]), {i,1,d}]]+a+Exp[1];
```

We define a simulator with parameters x_1 to x_{15} where only x_1 and x_2 are really used.

```
Simulator[x1_, x2_, x3_, x4_, x5_,
          x6_, x7_, x8_, x9_, x10_,
          x11_, x12_, x13_, x14_,
          x15_]:= f[{x1,x2}, 20, 0.2, 2*Pi, 2];
```

A plot of the ACKLEY function:

```
Plot3D[
    f[{x1,x2},20,0.2,2*Pi,2],
    {x1,-5,5},{x2,-5,5},
    PlotRange→Full, PlotPoints→100,
    AxesLabel→{"x1","x2","f(x1,x2)"},
    BaseStyle→{FontWeight→"Bold",FontSize→12},
    ColorFunction→"DarkRainbow",AspectRatio→1]
```

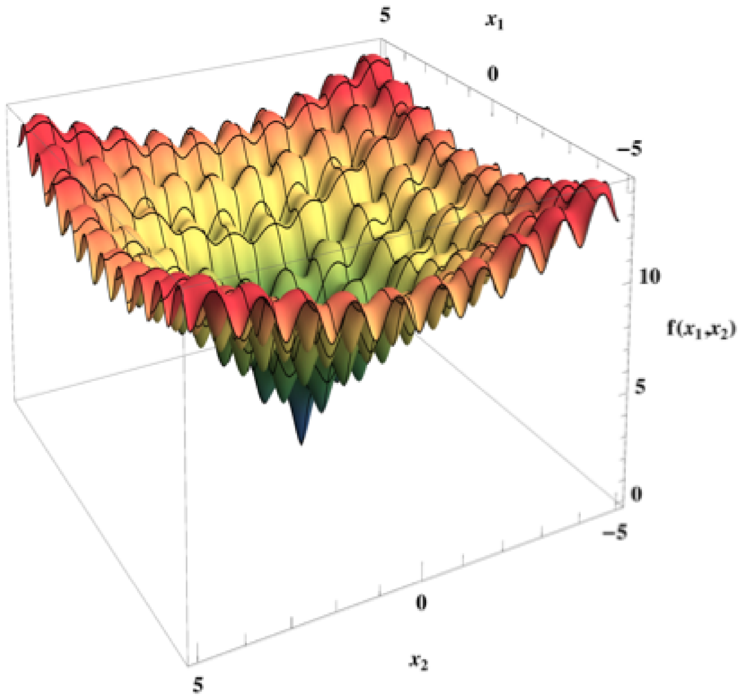

1. Design of experiment

Select an initial design with 50 points for 15 parameters:

```
SetDirectory[NotebookDirectory[]]  ;(** Set the working directory **)
spaceFillingDesign=Import[
        "Design15D_50points.csv",
        "FieldSeparators"→{","}
    ];
```

Use a good space filling design generator or a precomputed design.

Scale the initial design [0,1] to fit the parameter ranges of the simulator:

We use values for x_1 to x_{15} in]-5, 5[.

```
initialDesign=spaceFillingDesign/5-5;
ListPlot[initialDesign[[All,{1,2}]], FrameLabel→{x₁,x₁}, Frame→True]
```

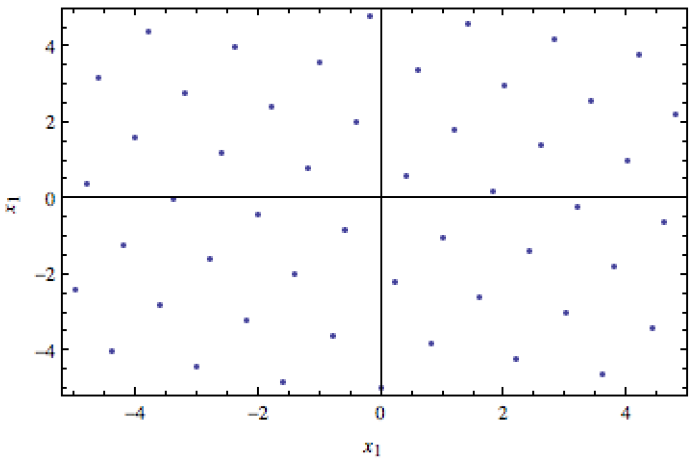

2. Simulation model

Run the simulator with the input combinations from the design:

```
simulatorOutput=Table[Apply[Simulator,x], {x,initialDesign}];
```

Join the input parameters with the simulator output:

```
initialDataSet=MapThread[Join[#1,{#2}]&,{initialDesign,simulatorOutput}];
```

The simulator output:

```
ListPlot3D[
    initialDataSet[[All,{1,2,16}]],
    PlotRange→Full, AxesLabel→{"x₁","x₂","f(x₁,x₂)"},
    BaseStyle→{FontWeight→"Bold",FontSize→12},
    ColorFunction→"DarkRainbow",AspectRatio→1]
```

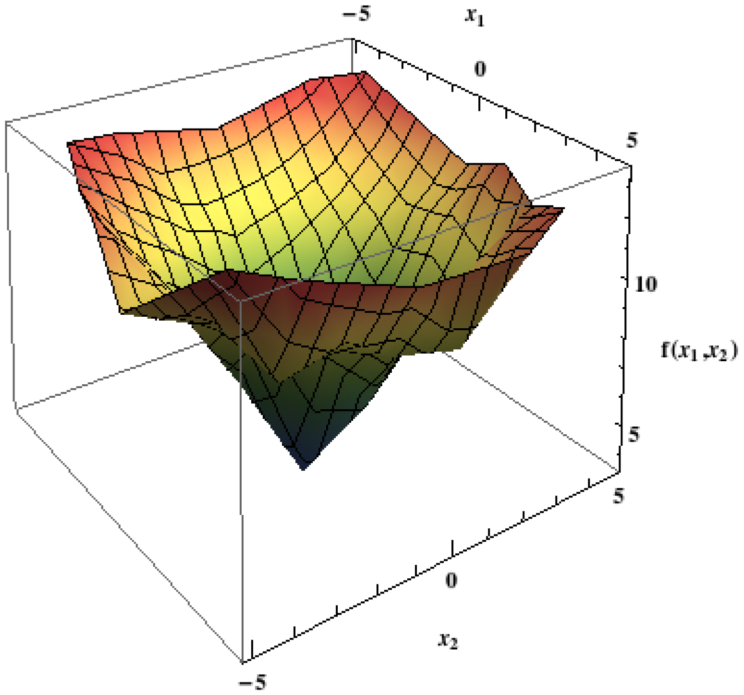

3. Surrogate modeling

The DataModeler package is required for the symbolic regression function that we use. See “http://www.evolved-analytics.com/?q=datamodeler” for more information.

Load the DataModeler package:

```
<<DataModeler`
modelingRunName1="RUN1";
```

Create surrogate models with symbolic regression:

```
modelSet1=
    SymbolicRegression[
            initialDataSet,16, (** The dataset, column 16 contains the response variab
            TimeConstraint→500, (** Do 500 seconds of modeling **)
            FunctionPatterns→{1,BuildFunctionPatterns["PowerMath","Sinusoids"]}
                                (** We will allow power functions and sinusoids **)
        ];
```

4. System understanding

▪ Pareto Front Log Plot

Plot the surrogate models. The vertical axis shows the model accuracy and the horizontal axis shows the complexity.

```
ParetoFrontLogPlot[modelSet1,PlotLabel→LabelForm@modelingRunName1]
```

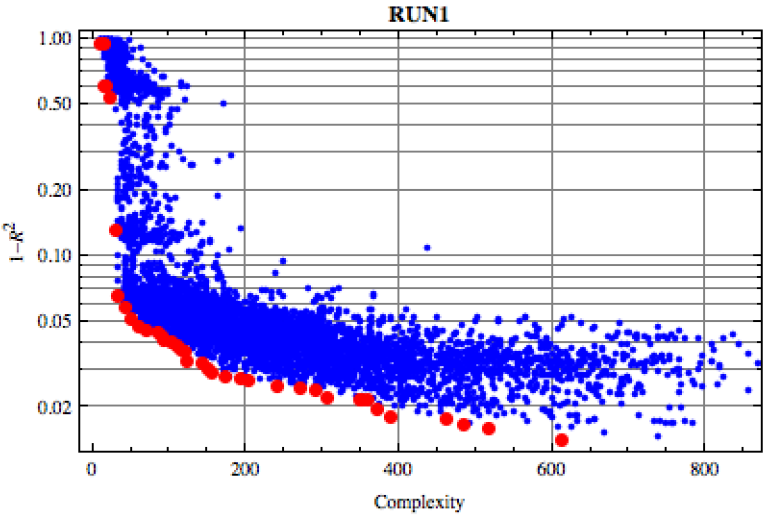

▪ Selecting high quality models

Only models with high accuracy and low complexity are used for predictions.

```
highQualityModels1=SelectModels[modelSet1,QualityBox→{650,0.04}];
```

```
ParetoFrontLogPlot[
    highQualityModels1,
    PlotLabel→LabelForm@(modelingRunName1<>" High Accuracy Models")]
```

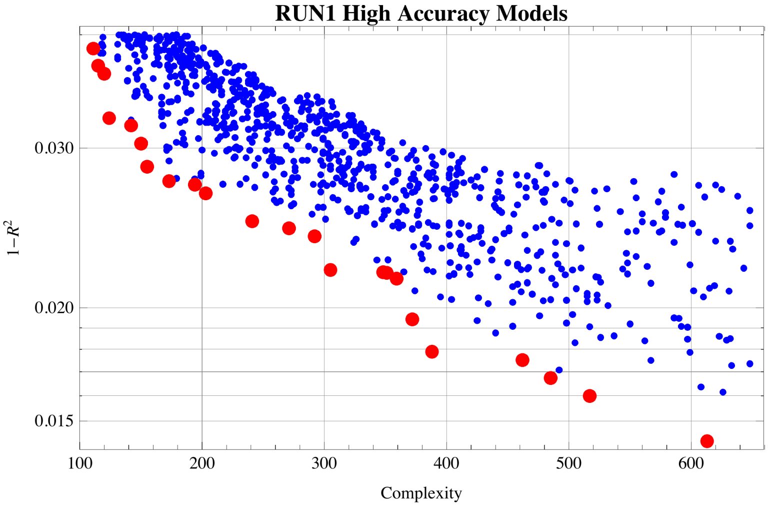

■ Feature Selection

Variable importance is related to variable presence in the high quality model ensemble.
In this example, only variables $x_1$and x_2 seem important. This is in accordance with the setup of our toy simulator.

```
VariableCombinationTable[modelSet1, SignificanceLevel→0.1]
```

Variable Combination Table

	num ⟹ %	Variables Used	ParetoFrontPlot
1	749 ⟹ 13.2 %	x1<br>x2	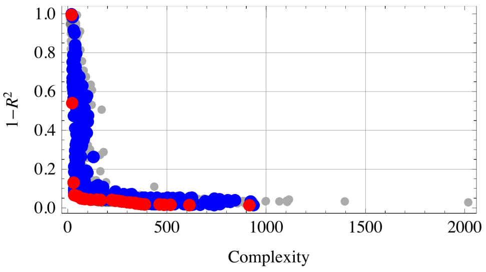

■ Create an ensemble for prediction

To make predictions, we need an "ensemble" data structure with only the robust models, without asymptotic behavior or discontinuities.

```
ensemble1=CreateModelEnsemble[RobustModels@highQualityModels1];
```

■ Observe the response surface behavior of the models

```
ResponsePlot[ensemble1,VariablesToPlot→{1,2}]
```

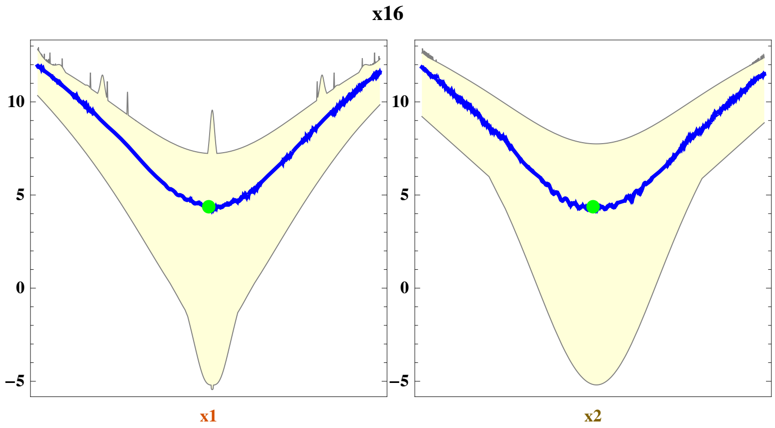

We observe large model disagreement in the global minimum of the function. This area requires more sampling in the next modeling iteration.

The predicted 3D surface with the surrogate models captures the general behavior of the simulator though refinements are needed.

```
ResponseSurfacePlot[
    ensemble1,
    VariablesToPlot→{1,2},
    PlotRange→Full, AxesLabel→{"x₁","x₂","f̃(x₁,x₂)"},
    BaseStyle→{FontWeight→"Bold",FontSize→12},
    ColorFunction→"DarkRainbow",AspectRatio→1, PlotLabel→None
]
```

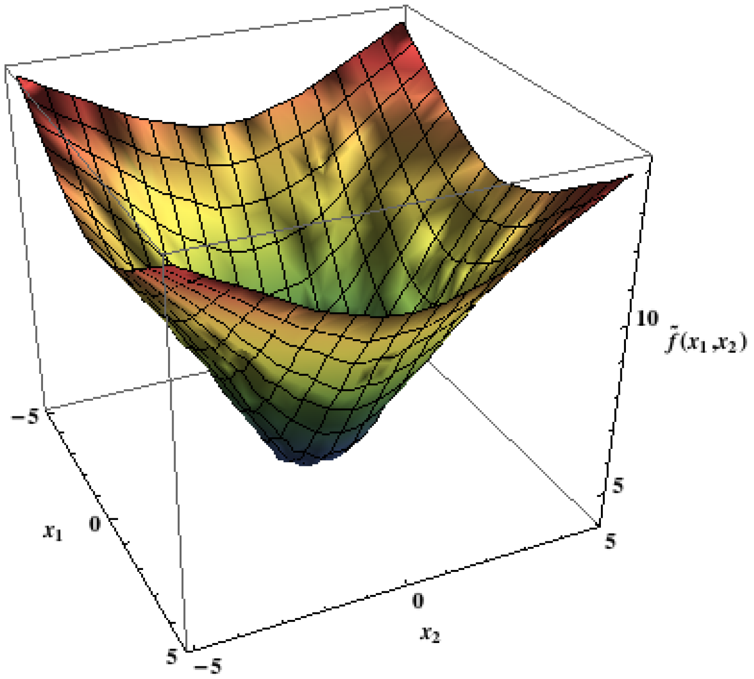

Next Iteration

1. Design of experiment

To improve the quality of our surrogate models we need to focus our design on $x_1$and x_2. We added 250 points to the original space filling design for $x_1$and x_2 homogeneously. It is also possible to sample especially the area of the predicted global minimum more into detail.

The new design:

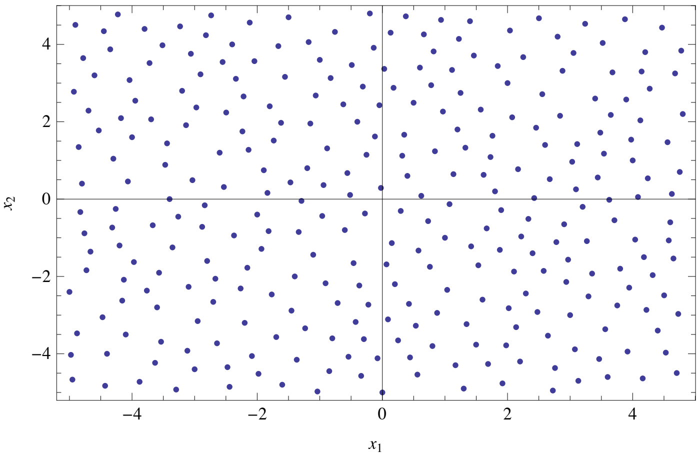

We repeated the previously described approach with the new input design...

2. Simulation model

...

3. Surrogate modeling

...

4. System understanding

- **The response surface behavior of the new surrogate models**

```
ResponsePlot[ensemble2,VariablesToPlot→{1,2}, PlotLabel→""]
```

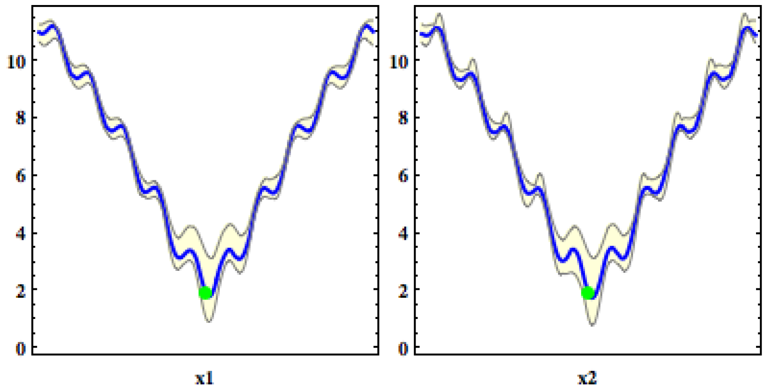

The model disagreement decreased and the behavior of the surrogate models represent the original simulator.

The response surface plot of the surrogate models:

```
ResponseSurfacePlot[
    ensemble2,
    VariablesToPlot→{1,2},
    PlotRange→Full, AxesLabel→{"x₁","x₂","f̃(x₁,x₂)"},
    BaseStyle→{FontWeight→"Bold",FontSize→12},
    ColorFunction→"DarkRainbow",AspectRatio→1, PlotLabel→None
]
```

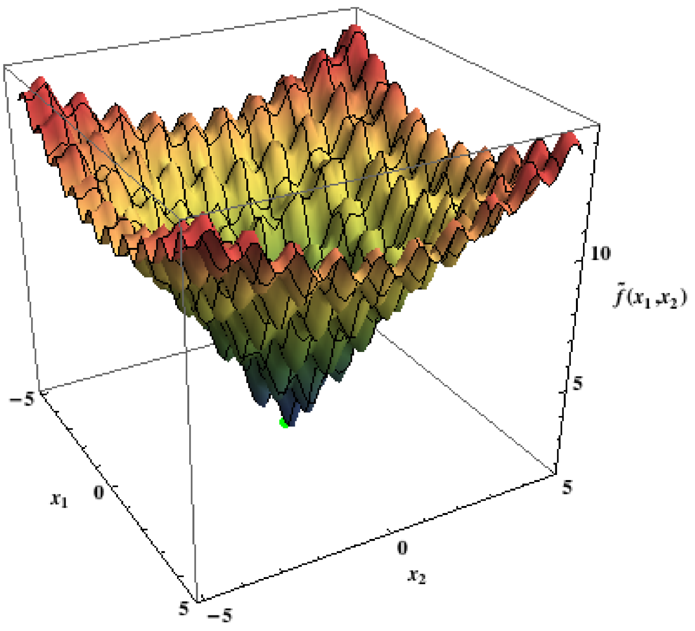

10
$\tilde{f}(x_1, x_2)$
5
−5
x_1
0
5
−5
0
x_2
5
