# Supplementary material for: Active Learning to Understand Infectious Disease Models and Improve Policy Making
Source: PLoS Comput Biol. 2014 Apr 17;10(4):e1003563. doi: 10.1371/journal.pcbi.1003563 (PMC3990517; doi:10.1371/journal.pcbi.1003563)
Supplement: Text S1 — Symbolic regression analysis FluTE. (PDF) [file pcbi.1003563.s002.pdf]

Text S1: Symbolic Regression Analyses FluTE

We performed five iterations with the FluTE model (parameters are listed in Table 2 from the main text) and analyzed the input-response data with SR. This supporting information contains a summary of the SR results. The SR parameters are presented in Table 1 in the main text. We present for each iteration:

1. Descriptive statistics for all obtained surrogate models and for the optimized high-quality ensemble.
2. The obtained surrogate models according complexity and model error and the optimized high-quality ensemble.
3. Surrogate model examples from the optimized high-quality ensemble for the AR and the peak day.

RUN 1

The first run with FluTE contained demographic data from Seattle and four transmission parameters. All surrogate models obtained with SR are presented according to complexity and model error in Figure S1. We selected models in the knee of the Pareto front to obtain a high-quality model ensemble with limited complexity. This ensemble is optimized using nonlinear techniques to end up with a predictive set, presented in Figure S1. Descriptive statistics for all surrogate models and for the optimized high-quality model are given in Table S1. Table S2 illustrates surrogate models for the AR and the epidemic peak day, arbitrary chosen from the Pareto front. The complete optimized high-quality model ensemble for the AR is given in Text S2.

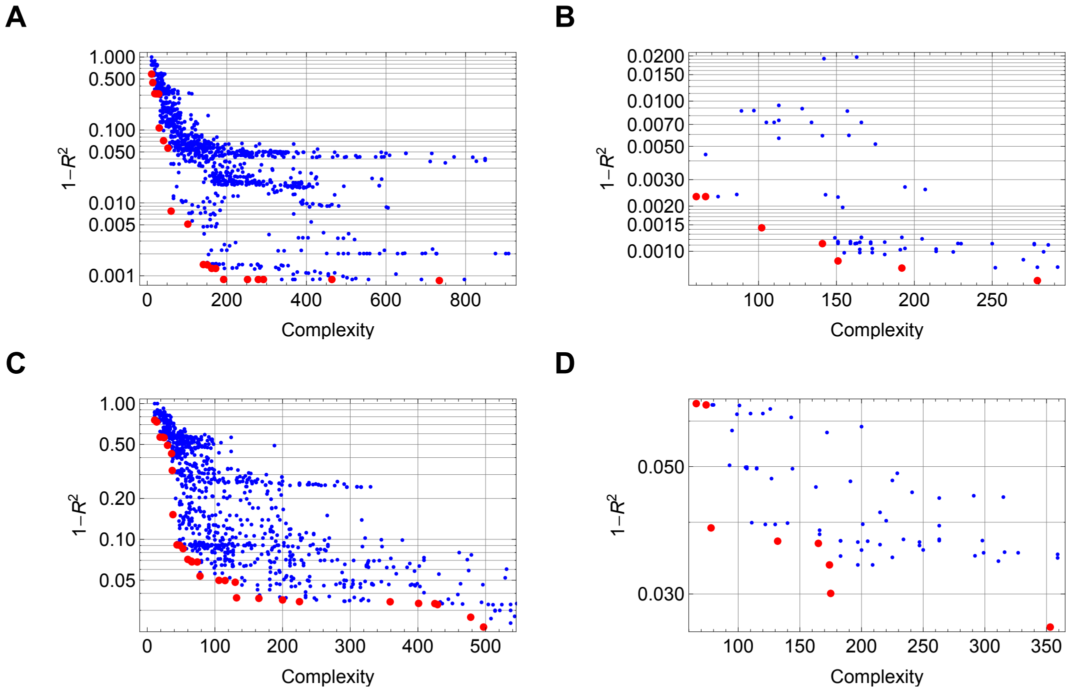

Figure S1. Model error (1-R^2) and complexity of the surrogate models for the AR and epidemic peak from RUN 1. The models indicated in red are on the Pareto front and the model error is presented on a log scale. A: AR, all models - B: AR, optimized high-quality ensemble - C: peak day, all models - D: peak day, optimized high-quality ensemble.

Table S1. Surrogate model set specifications from RUN 1 . The generation count represents the average number of generations from all independent evolutions.

**Response**	**Generation count**	**Max Model age**	**Mean Model Age**	**Min Model Error**	**Mean Model Complexity**
AR	232	139	32	0.000860588	186
AR (ensemble)	-	122	45	0.000638384	171
Peak Day	227	130	29	0.022570700	140
Peak Day (ensemble)	-	130	38	0.026274200	191

Table S2. Surrogate model examples from RUN 1. The models are arbitrary chosen from the Pareto front of the optimized high-quality ensemble from RUN 1

**Response**	**Complexity**	**1-R^2**	**Function**
AR	60	0.002	$\frac{-0.737}{\frac{-2.899}{3.450+\text{Seedinfected}}+\text{Seedinfected*SeedInfectedDaily}\left(6.015\times10^{-4}\right)+\text{R0}}+0.799$
Peak Day	78	0.039	$\frac{54.305}{\frac{1}{-0.025+\text{Seedinfected}\left(-4.202\times10^{12}\right)}+0.029\sqrt{\text{Seedinfected}}-1.007+\text{R0}+\text{SeedInfectedDaily}}+1.429$

RUN 2

The second FluTE run was again based on demographic data from Seattle and four transmission parameters but the seeding parameter was different. We sampled low seeding values more into detail to include more edge cases from the simulation model and increase stochasticity of the results. All surrogate models obtained with SR are presented according to complexity and model error in Figure S2. We selected models in the knee of the Pareto front to obtain a high-quality model ensemble with limited complexity. This ensemble is optimized using nonlinear techniques and is presented in Figure S2. Descriptive statistics for all surrogate models and for the optimized high-quality model are given in Table S3. Table S4 illustrates surrogate models for the AR and the epidemic peak day. We present two model examples for the AR to illustrate the trade-off between model error and complexity.

Table S3. Surrogate model set specifications from RUN 2 . The generation count represents the average number of generations from all independent evolutions.

**Response**	**Generation count**	**Max Model age**	**Mean Model Age**	**Min Model Error**	**Mean Model Complexity**
AR	231	173	36	0.159324	184
AR (ensemble)	-	165	50	0.158718	104
Peak Day	221	181	41	0.319475	156
Peak Day (ensemble)	-	130	38	0.0262742	191

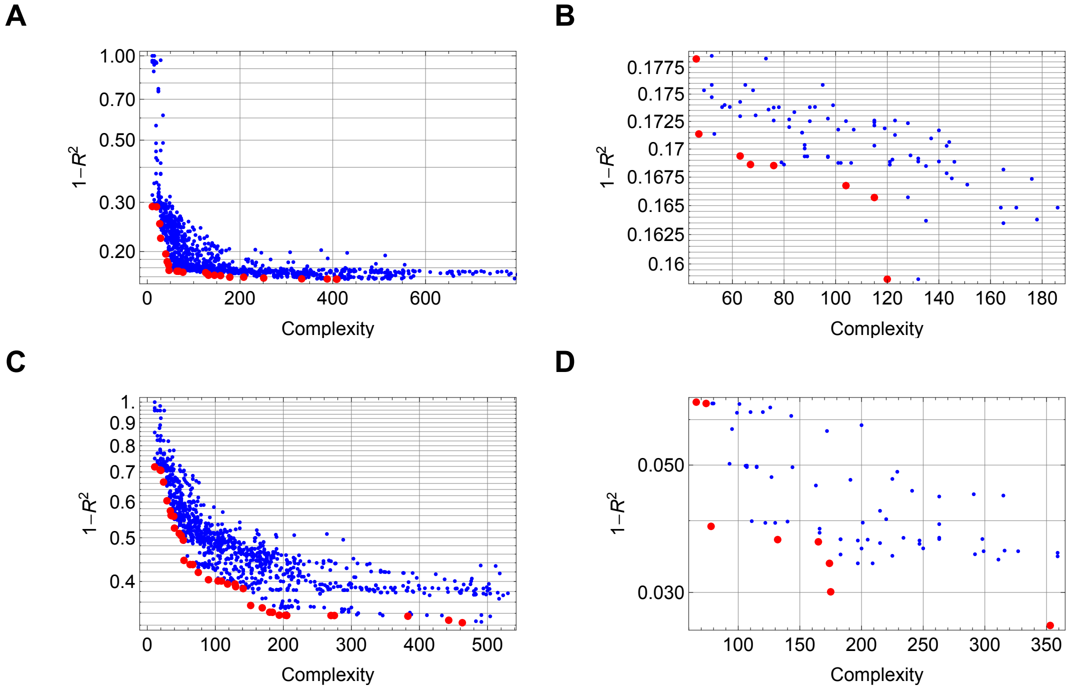

Figure S2. Model error (1-R^2) and complexity of the surrogate models for the AR and epidemic peak from RUN 2. The models indicated in red are on the Pareto front and the model error is presented on a log scale. A: AR, all models - B: AR, optimized high-quality ensemble - C: peak day, all models - D: peak day, optimized high-quality ensemble.

Table S4. Surrogate model examples from RUN 2. The models are arbitrary chosen from the Pareto front of the optimized high-quality ensemble from RUN 2

**Response**	**Complexity**	**1-R^2**	**Function**
AR	63	0.169	$0.931+\frac{-6.549}{\frac{-1.067}{\mathrm{Seedinfected}}+5.591\mathrm{R0}+\sqrt[8]{\mathrm{Seedinfected}}+\mathrm{SeedInfectedDaily}}$
AR	120	0.159	$\frac{-2.571\mathrm{R0}^{-0.339}3.612^{-0.818\mathrm{R0}}\left(3.794+\frac{\mathrm{R0}}{\mathrm{Seedinfected}}\right)}{0.004\mathrm{Seedinfected}(0.217+\mathrm{SeedInfectedDaily})+5.561+\mathrm{SeedInfectedDaily}}+0.593$
Peak Day	78	0.039	$\frac{54.305}{\frac{1}{-0.025+\mathrm{Seedinfected}\left(-4.202\times10^{12}\right)}+0.029\sqrt{\mathrm{Seedinfected}}-1.007+\mathrm{R0}+\mathrm{SeedInfectedDaily}}+1.429$

RUN 3

The third FluTE run was based on demographic data from LA county and four transmission parameters with the low seeding numbers. All surrogate models obtained with SR are presented according to complexity and model error in Figure S3. We selected models in the knee of the Pareto front to obtain a high-quality model ensemble with limited complexity. This ensemble is optimized using nonlinear techniques to end up with a predictive set, presented in Figure S3. We performed SR analysis with two time budgets, 1000s and 2400s and obtained similar results. We observe a very small decrease in model error at a high computational cost to find more complex functions. Descriptive statistics for all surrogate models and for the optimized high-quality model from the 1000s and 2400s analysis are given in Table S5. Table S6 illustrates surrogate model examples for the AR and epidemic peak day.

Table S5. Surrogate model set specifications from RUN 3 . The generation count represents the average number of generations from all independent evolutions.

Response	Generation count	Max Model age	Mean Model Age	Min Model Error	Mean Model Complexity
AR (1000s)	773	542	89	0.177380	245
AR (2400s)	-	1286	171	0.175891	327
AR (1000s, ensemble)	-	401	102	0.175269	198
AR (2400s, ensemble)	-	828	259	0.174229	320
Peak Day	693	355	80	0.412795	247
Peak Day (ensemble)	-	340	113	0.425443	136

Table S6. Surrogate model examples from RUN 3. The models are arbitrary chosen from the Pareto front of the optimized high-quality ensemble from RUN 3 and contain SeedInfected (a) and R_0 (b)

Response	Complexity	1-R^2	Function
AR	187	0.178	$0.233\left(-0.162B+\frac{-0.714}{A}+\frac{-0.921}{15.001-2.554a+a^{b}+b}+\frac{1}{\frac{1}{-0.983a+b}+9.653+b}+\frac{-4.092}{b}\right)+0.963$
Peak Day	85	0.431	$\frac{100.767}{1.819b-3.367+\log(\log(4.064a))+\frac{1}{a\log(b)}}+13.869$

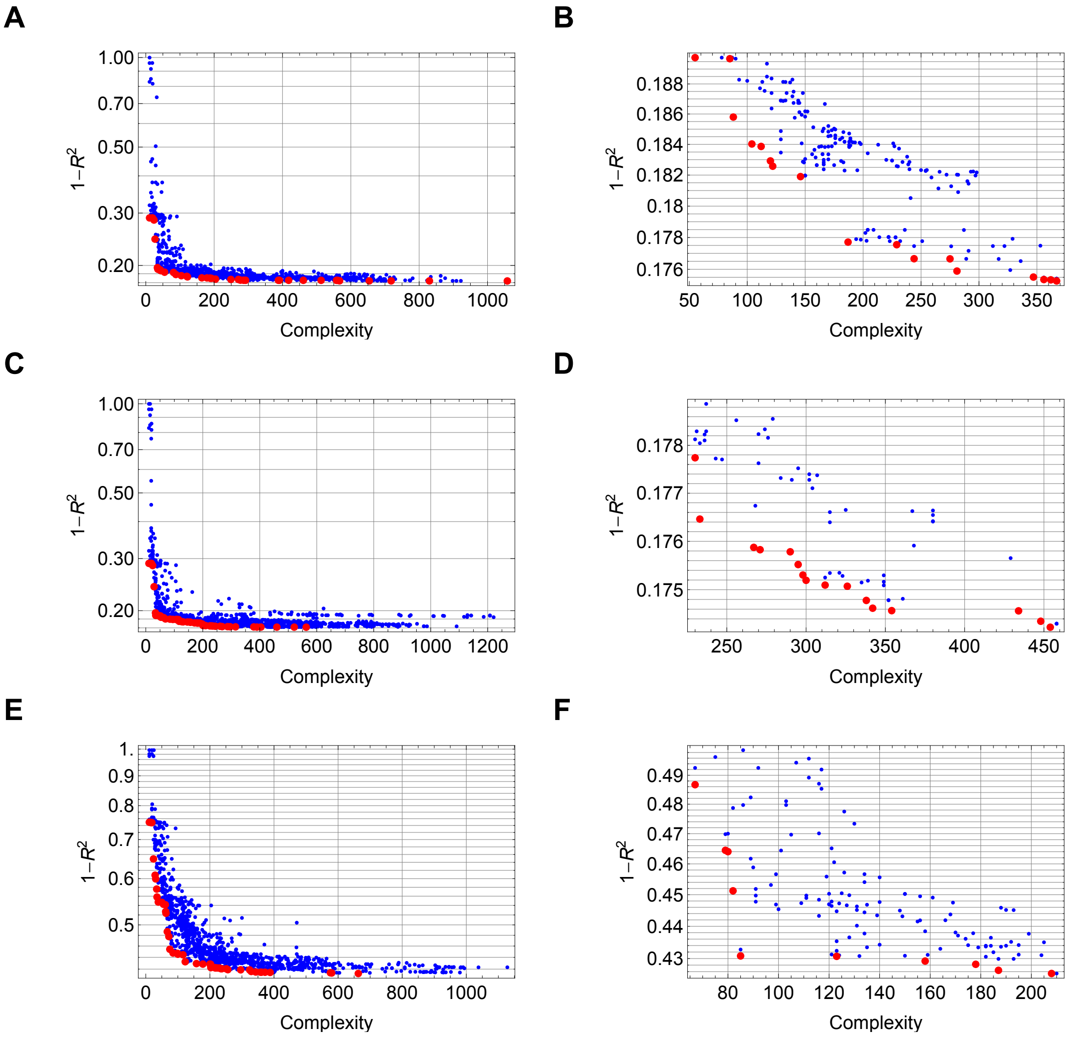

Figure S3. Model error (1-R^2) and complexity of the surrogate models for the AR and epidemic peak from RUN 3. The models indicated in red are on the Pareto front and the model error is presented on a log scale. A: AR, all models after 1000s - B: AR, optimized high-quality ensemble after 1000s - C: AR, all models after 2400s - D: AR, optimized high-quality ensemble after 2400s - E: peak day, all models - F: peak day, optimized high-quality ensemble.

RUN 4

The fourth FluTE run was performed with demographic data from Seattle, four transmission parameters with the low seeding numbers and eight vaccination parameters. All surrogate models obtained with SR are presented according to complexity and model error in Figure S4. We selected models in the knee of the Pareto front to obtain a high-quality model ensemble with limited complexity. This ensemble is optimized using nonlinear techniques to end up with a predictive set, presented in Figure S4. Descriptive statistics for all surrogate models and for the optimized high-quality model are given in Table S7. Table S8 illustrates surrogate models for the AR and epidemic peak day.

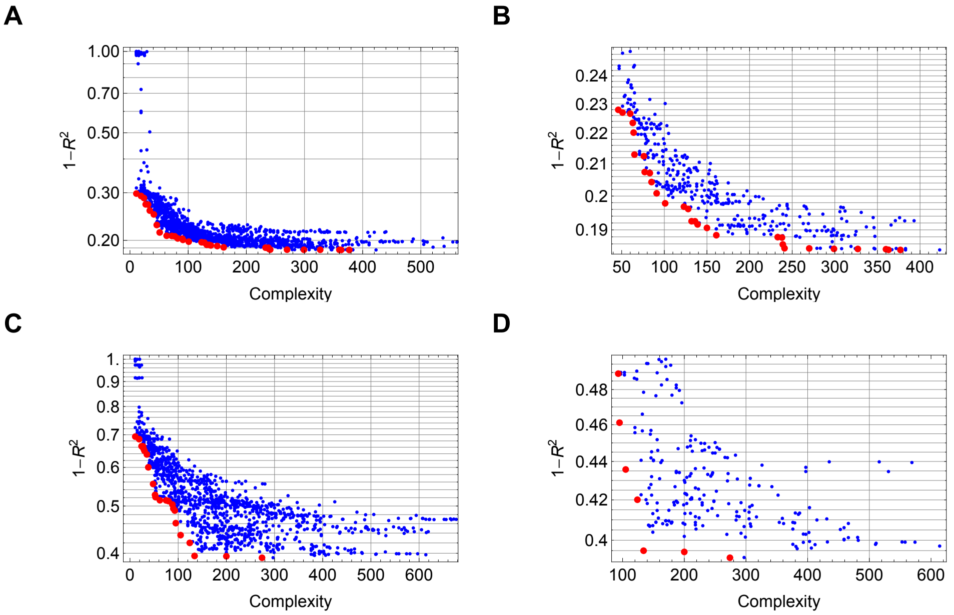

Figure S4. Model error (1-R^2) and complexity of the surrogate models for the AR and epidemic peak from RUN 4. The models indicated in red are on the Pareto front and the model error is presented on a log scale. A: AR, all models - B: AR, optimized high-quality ensemble - C: peak day, all models - D: peak day, optimized high-quality ensemble.

Table S7. Surrogate model set specifications from RUN 4 . The generation count represents the average number of generations from all independent evolutions.

Response	Generation count	Max Model age	Mean Model Age	Min Model Error	Mean Model Complexity
AR	416	240	51	0.184299	152
AR (ensemble)	-	203	57	0.184299	164
Peak Day	441	254	57	0.391609	188
Peak Day (ensemble)	-	203	77	0.391609	251

Table S8. Surrogate model examples from RUN 4. The models are arbitrary chosen from the Pareto front of the optimized high-quality ensemble from RUN 4 and contain SeedInfected (a), R_0 (b), SeedInfectedDaily (c), Travel (d), AscertainmentFraction (e), AscertainmentDelay (f), ResponseThreshold (g), VaccinationCoverage (h),VEsusceptibility (i)

Response	Complexity	1-R^2	Function
AR	161	0.188	$0.823 + \frac{-16.853\left(-1.048g+\frac{1}{b}\right)}{\frac{-12.507}{-1.674i+1.964+6.888c+a+\log(a)}-1.623e+18.609+\sqrt{f}+c+d}$
Peak Day	134	0.395	$\frac{464.427}{b\left(1.434+\sqrt{a}+h\right)\left(3.243^{\frac{2.002}{c\left(1.522\times 10^{6}\right)+a}}-1.754+b+c\right)} + 26.035$

RUN 5

The fifth FluTE run was performed with demographic data from LA, four transmission parameters with the low seeding numbers and six vaccination parameters to model instant reaction strategies. All surrogate models obtained with SR are presented according to complexity and model error in Figure S5. We selected models in the knee of the Pareto front to obtain a high-quality model ensemble with limited complexity. This ensemble is optimized using nonlinear techniques to end up with a predictive set, presented in Figure S5. Descriptive statistics for all surrogate models and for the optimized high-quality models are given in Table S9. Table S10 illustrates surrogate models for the AR and the epidemic peak day.

Table S9. Surrogate model set specifications from RUN 5 . The generation count represents the average number of generations from all independent evolutions.

Response	Generation count	Max Model age	Mean Model Age	Min Model Error	Mean Model Complexity
AR	763	573	97	0.115576	230
AR (ensemble)	-	570	138	0.105285	229
Peak Day	751	500	92	0.349411	238
Peak Day (ensemble)	-	91	31	0.412062	291

Table S10. Surrogate model examples from RUN 5. The models are arbitrary chosen from the Pareto front of the optimized high-quality ensemble for RUN 5 and contain SeedInfected (a), R_0 (b), VaccinationCoverage (c), VEsusceptibility (d) and VEinfectiousness (e)

Response	Complexity	1-R^2	Function
AR	150	0.136	$-0.003 + 0.579(0.959 + -1.085cd + -1.255ce)2.918^{\frac{-0.814}{b^2 \log(b)(-1.120c+1.122)}}$
PeakDay	197	0.404	$\frac{251.047}{\left(-1.041b+6.153^{-1.238b+2.641^{c}+bcd+e}+\frac{4.672}{a}\right)^2+b+\frac{1}{a}+d^2} + 17.969$

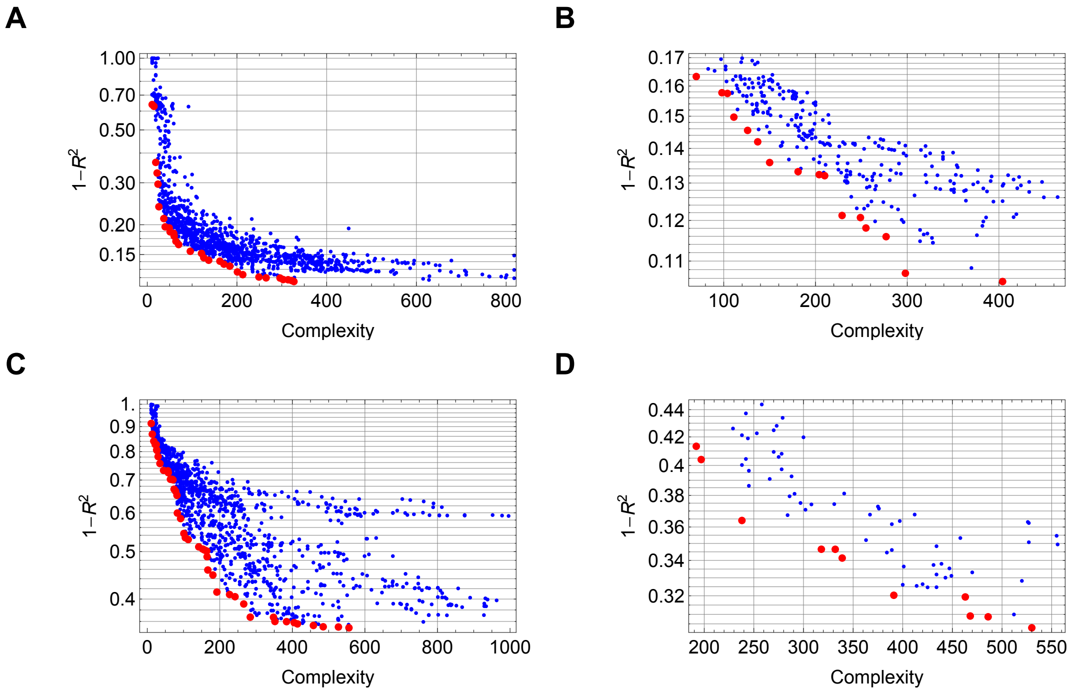

Figure S5. Model error (1-R^2) and complexity of the surrogate models for the AR and epidemic peak from RUN 5. The models indicated in red are on the Pareto front and the model error is presented on a log scale. A: AR, all models - B: AR, optimized high-quality ensemble - C: peak day, all models - D: peak day, optimized high-quality ensemble.
