# Supplementary material for: Active Learning to Understand Infectious Disease Models and Improve Policy Making
Source: PLoS Comput Biol. 2014 Apr 17;10(4):e1003563. doi: 10.1371/journal.pcbi.1003563 (PMC3990517; doi:10.1371/journal.pcbi.1003563)
Supplement: Text S2 — Optimized high-quality model ensemble RUN 1. (PDF) [file pcbi.1003563.s003.pdf]

Text S2: Optimized High-Quality Model Ensemble RUN 1

Table S1. Optimized high-quality surrogate model ensemble for the AR from RUN 1 with SeedInfected (a), R_0 (b) and SeedInfectedDaily (c).

Complexity	1-$\mathbf{R}^2$	Function
60	0.002	$\frac{-0.737}{\frac{-2.899}{3.450+a}+ac\left(6.015\times10^{-4}\right)+b}+0.799$
66	0.002	$\frac{-0.740}{\frac{2.283\times10^{-10}}{-1.763+b+a}+ac\left(6.012\times10^{-4}\right)+b}+0.800$
66	0.004	$\frac{-0.748}{\frac{8.837}{-11.274+a+c}+ac\left(6.021\times10^{-4}\right)+b}+0.801$
74	0.002	$\frac{-0.737}{\frac{32204.600}{-11108.500a-38325.800}+ac\left(6.015\times10^{-4}\right)+b}+0.799$
86	0.002	$-0.505\left(\frac{1.464}{ac\left(6.005\times10^{-4}\right)+b}+\frac{1}{\sqrt{b}+a}\right)+0.800$
89	0.009	$0.697+\frac{-2.389}{\left(\frac{a}{10.596+a}+ac\left(6.620\times10^{-4}\right)+b\right)^2}$
97	0.009	$0.697+\frac{-2.389}{\left(ac\left(6.614\times10^{-4}\right)+\frac{a}{9.058+b+a}+b\right)^2}$
102	0.001	$0.638+\frac{-4.405}{-0.644+2.721^{\frac{a}{0.719+a}+ac\left(6.184\times10^{-4}\right)+b}+b}$
105	0.007	$0.619-3.777\exp\left(ac\left(-7.849\times10^{-4}\right)-b-e^{-\sqrt{\frac{1}{a}}}\right)$
110	0.007	$0.619-11258.800\exp\left(ac\left(-7.849\times10^{-4}\right)-b-e^{-\sqrt{\frac{1}{a}}}-8\right)$
113	0.006	$0.618+\frac{-3.758}{-1.494+\exp\left(ac\left(7.849\times10^{-4}\right)+b+e^{-\sqrt{\frac{1}{a}}}\right)+b}$
113	0.007	$0.628+\frac{-4.454}{\exp\left(ac\left(7.849\times10^{-4}\right)+b+e^{-\sqrt{\frac{1}{a}}}\right)+7.849\times10^{-4}+b}$
113	0.009	$0.620+\frac{-3.851}{\exp\left(ac\left(7.849\times10^{-4}\right)+b+e^{-\sqrt{\frac{1}{a}}}\right)+7.849\times10^{-4}+c}$
128	0.009	$-3.538\exp\left(\exp\left(ac\left(-7.849\times10^{-4}\right)-b-e^{-\sqrt{\frac{1}{a}}}\right)\right)+4.153$
134	0.007	$-11258.600\exp\left(\exp\left(ac\left(-7.849\times10^{-4}\right)-b-e^{-\sqrt{\frac{1}{a}}}-8\right)\right)+11259.200$
141	0.001	$\frac{-88.811}{\left(-0.028+5.393\left(\frac{a}{3.037+a}+ac\left(7.285\times10^{-4}\right)+b\right)+b\right)^2}+0.702$
141	0.006	$-0.203\left(\frac{1.853}{0.011c(0.020a+b)+\sqrt{b}}+\frac{1}{b+a}\right)^2+0.772$
142	0.019	$\frac{-109295\exp\left(e^{-0.253a}-3.833-b\right)}{1.081\sqrt{a^2c^2}+1484.140+b}+0.665$
143	0.002	$-0.367\left(\frac{2.015}{ac\left(6.007\times10^{-4}\right)+b}+\frac{1}{\sqrt{\frac{1}{\sqrt[8]{b}+a}+a}}\right)+0.800$
149	0.001	$\frac{2.844\left(\frac{3.075}{2.887+\sqrt{b}}+\frac{1}{\sqrt{b}+a}\right)}{-0.002ac^2-3.176b+ac\left(-7.586\times10^{-4}\right)}+0.753$
151	8.624×10^{-4}	$\frac{0.867\left(\frac{1.509}{0.501+\sqrt{b}}+\frac{1}{\sqrt[4]{b}+a}\right)}{-0.340-0.929b+ac^2\left(-9.338\times10^{-4}\right)}+0.742$
151	0.001	$\frac{-88.223}{\left(-0.035+5.376\left(\frac{a}{1.489+b+a}+ac\left(7.283\times10^{-4}\right)+b\right)+b\right)^2}+0.702$
151	0.001	$\frac{-88.196}{\left(-0.037+5.377\left(\frac{a}{2.596+a+c}+ac\left(7.298\times10^{-4}\right)+b\right)+b\right)^2}+0.702$

continued on next page

Table S1 – continued from previous page (a: SeedInfected, b: R_0 and c: SeedInfectedDaily)

Complexity	1-R^2	Function
151	0.001	$\frac{-87.310}{\left(-0.028+5.339\left(\mathrm{ac}\left(7.297\times10^{-4}\right)+\frac{\mathrm{a}}{\mathrm{b+a+c}}+\mathrm{b}\right)+\mathrm{b}\right)^2}+0.702$
151	0.002	$0.710+\frac{-322.488}{\left(10.680\left(\mathrm{ac}\left(6.140\times10^{-4}\right)+\frac{\mathrm{a}}{\mathrm{b+a+c}}+\mathrm{b}\right)+\mathrm{b}+\mathrm{c}\right)^2}$
154	0.002	$0.696+\frac{-75.710}{\left(5\left(\mathrm{ac}\left(7.849\times10^{-4}\right)+\mathrm{b}+e^{-\sqrt{\frac{1}{\mathrm{a}}}}\right)+7.849\times10^{-4}+\mathrm{b}\right)^2}$
155	9.774×10^{-4}	$\frac{-110.383}{\left(0.182\mathrm{c}+6.043\left(\mathrm{ac}\left(6.927\times10^{-4}\right)+\frac{\mathrm{a}}{4.702+\mathrm{a}}+\mathrm{b}\right)+\mathrm{b}\right)^2}+0.705$
157	0.009	$0.697+\frac{-2.388}{\left(\frac{\mathrm{a}}{10.531+\frac{\mathrm{a}}{\mathrm{a}\left(-8.600\times10^{6}\right)+6.140\times10^{9}}+\mathrm{a}}+\mathrm{ac}\left(6.619\times10^{-4}\right)+\mathrm{b}\right)^2}$
158	0.006	$-4.163\exp\left(\frac{1}{-0.140+\exp\left(\mathrm{ac}\left(7.849\times10^{-4}\right)+\mathrm{b}+e^{-\sqrt{\frac{1}{\mathrm{a}}}}\right)+\mathrm{b}}\right)+4.787$
159	0.001	$0.703+\frac{-71.711}{\left(0.895\mathrm{b}+4.834\left(\frac{\mathrm{a}}{1.443+\mathrm{b}+\mathrm{a}}+\mathrm{ac}\left(7.271\times10^{-4}\right)+\mathrm{b}\right)\right)^2}$
159	0.001	$0.703+\frac{-107.726}{\left(1.104\mathrm{b}+5.918\left(\mathrm{ac}\left(7.287\times10^{-4}\right)+\frac{\mathrm{a}}{\mathrm{b+a+c}}+\mathrm{b}\right)\right)^2}$
162	0.001	$\frac{-89.867}{\left(5.414\left(0.011\mathrm{ac}+\frac{\mathrm{a}}{3+\mathrm{a}}+\mathrm{b}\right)-0.057\mathrm{ac}+\mathrm{b}\right)^2}+0.703$
163	0.001	$\frac{-87.680}{\left(-0.037+5.357\left(\frac{\mathrm{a}}{1.829\mathrm{b}+\mathrm{a}}+\mathrm{ac}\left(7.281\times10^{-4}\right)+\mathrm{b}\right)+\mathrm{b}\right)^2}+0.702$
163	0.020	$0.013\log\left(0.306\mathrm{a}\left(0.306+\mathrm{b}^{4.625}+\sqrt{\mathrm{bac}}+\mathrm{b}-\mathrm{c}\right)^{9.249}+40.635\right)-0.058$
165	9.801×10^{-4}	$\frac{-109.989}{\left(0.182\mathrm{c}+6.030\left(\frac{\mathrm{a}}{3.149+\mathrm{b}+\mathrm{a}}+\mathrm{ac}\left(6.921\times10^{-4}\right)+\mathrm{b}\right)+\mathrm{b}\right)^2}+0.706$
165	0.001	$\frac{-104.291}{\left(0.158\mathrm{c}+5.851\left(\mathrm{ac}\left(6.984\times10^{-4}\right)+\frac{\mathrm{a}}{\mathrm{b+a+c}}+\mathrm{b}\right)+\mathrm{b}\right)^2}+0.705$
165	0.001	$0.703+\frac{-84.771}{\left(5.230\left(\mathrm{ac}\left(7.332\times10^{-4}\right)+\frac{\mathrm{a}}{\mathrm{b+a+c}}+\mathrm{b}\right)+\mathrm{a}\left(8.899\times10^{-6}\right)+\mathrm{b}\right)^2}$
166	0.001	$\frac{14.449\left(\frac{3.558}{2.901+\sqrt{\mathrm{b}}}+\frac{1}{\sqrt[4]{\mathrm{b}}+\mathrm{a}}\right)}{0.022\mathrm{ac}-0.036\mathrm{ac}^2-18.599\mathrm{b}}+0.753$
166	0.001	$\frac{3.182\left(\frac{3.826}{2.907+\sqrt{\mathrm{b}}}+\frac{1}{\sqrt[8]{\mathrm{b}}+\mathrm{a}}\right)}{0.113\mathrm{ac}-0.117\mathrm{ac}^2-4.397\mathrm{b}}+0.753$
166	0.007	$-11259.300\exp\left(\frac{1}{-0.140+\exp\left(\mathrm{ac}\left(7.849\times10^{-4}\right)+\mathrm{b}+e^{-\sqrt{\frac{1}{\mathrm{a}}}}+8\right)+\mathrm{b}}\right)+11259.900$
172	0.001	$\frac{-89.544}{\left(5.402\left(0.002\mathrm{ac}+\frac{\mathrm{a}}{1.443+\mathrm{b}+\mathrm{a}}+\mathrm{b}\right)-0.009\mathrm{ac}+\mathrm{b}\right)^2}+0.703$
172	0.001	$\frac{-89.595}{\left(5.405\left(0.123\mathrm{ac}+\frac{\mathrm{a}}{2.546+\mathrm{a}+\mathrm{c}}+\mathrm{b}\right)-0.663\mathrm{ac}+\mathrm{b}\right)^2}+0.703$
173	9.801×10^{-4}	$\frac{-109.989}{\left(0.182\mathrm{c}^2+6.030\left(\frac{\mathrm{a}}{3.149+\mathrm{b}+\mathrm{a}}+\mathrm{ac}\left(6.921\times10^{-4}\right)+\mathrm{b}\right)+\mathrm{b}\right)^2}+0.706$
173	0.001	$\frac{-313.318}{\left(0.340\mathrm{b}^2+10.426\left(\frac{\mathrm{a}}{2.848+\mathrm{a}+\mathrm{c}}+\mathrm{ac}\left(7.479\times10^{-4}\right)+\mathrm{b}\right)+\mathrm{b}\right)^2}+0.695$
175	0.005	$0.680+\frac{-73.045}{\left(5\left(\mathrm{ac}\left(7.849\times10^{-4}\right)+\mathrm{b}+e^{-\sqrt{\frac{1}{\mathrm{a}}}}\right)+\mathrm{ac}\left(7.849\times10^{-4}\right)+\mathrm{b}\right)^2}$
181	9.525×10^{-4}	$\frac{-233.224}{\left(0.110\mathrm{b}^3+9.046\left(\frac{\mathrm{a}}{2.760+\mathrm{a}+\mathrm{c}}+\mathrm{ac}\left(7.559\times10^{-4}\right)+\mathrm{b}\right)+\mathrm{b}\right)^2}+0.693$

continued on next page

Table S1 – continued from previous page (a: SeedInfected, b: R_0 and c: SeedInfectedDaily)

Complexity	1-R^2	Function
181	0.001	$0.703+\frac{-84.765}{\left(0.970b+5.261\left(\frac{a}{\frac{3.892}{b}+a+c}+ac\left(7.293\times10^{-4}\right)+b\right)\right)^2}$
191	0.001	$0.699+\frac{-91.273}{\left(5.484\left(\frac{a}{1.722b+a}+ac\left(6.459\times10^{-4}\right)+b\right)+bac\left(3.472\times10^{-4}\right)+b\right)^2}$
192	7.733×10^{-4}	$0.707+\frac{-87.716}{\left(a^2c\left(-1.433\times10^{-7}\right)+5.306\left(\frac{a}{2.468b+a}+ac\left(7.823\times10^{-4}\right)+b\right)+b\right)^2}$
192	0.001	$\frac{0.723\left(\frac{3.828}{2.909+\sqrt{b}}+\frac{1}{\sqrt[8]{b}+a}\right)}{-1b+c\left(-1.450\times10^{-4}\right)(2.501a+Travel)+ac^2\left(-3.951\times10^{-4}\right)}+0.753$
194	0.001	$\frac{-91.636}{\left(5.474\left(0.025ac+\frac{a}{6.255-6.752c+a}+b\right)-0.132ac+b\right)^2}+0.703$
194	0.003	$-0.010\left(\frac{11.236+\frac{81}{\left(ac\left(3.931\times10^{-4}\right)+\sqrt{b}\right)^2}}{0.291+\sqrt{b}}+\frac{29.120}{\sqrt{0.516+a}}\right)+0.745$
205	0.001	$\frac{0.725\left(\frac{4.202}{3.305+\sqrt{b}}+\frac{1}{\sqrt[8]{b}+a}\right)}{-0.993b+c\left(7.231\times10^{-6}\right)\left(-2378+3.599a+\sqrt{b}\right)+ac^2\left(-7.523\times10^{-4}\right)}+0.758$
207	0.003	$-0.010\left(\frac{\frac{81}{\left(ac\left(3.931\times10^{-4}\right)+\sqrt{b}\right)^2}+7.389+\sqrt{b}}{0.291+\sqrt{b}}+\frac{29.120}{\sqrt{0.516+a}}\right)+0.732$
214	9.994×10^{-4}	$0.705+\frac{-111.800}{\left(6.092\left(c(22.882b+a)\left(7.740\times10^{-4}\right)+\frac{a}{4.078+a+c}+b\right)+ac\left(-4.615\times10^{-4}\right)+b\right)^2}$
214	0.001	$0.704+\frac{-105.475}{\left(5.894\left(c(20.345b+a)\left(7.622\times10^{-4}\right)+\frac{a}{b+a+c}+b\right)+ac\left(-3.491\times10^{-4}\right)+b\right)^2}$
225	9.902×10^{-4}	$\frac{-111.882}{\left(6.090\left(0.013c\left(b+\frac{1}{\sqrt{b}}+a\right)+\frac{a}{4.227+a+c}+b\right)-0.073ac+b\right)^2}+0.705$
228	0.001	$\frac{-82.814}{\left(\frac{-0.673}{b\left(\frac{20.425}{b}+a+c\right)}+5.176\left(\frac{a}{1.427+b+a}+ac\left(7.327\times10^{-4}\right)+b\right)+b\right)^2}+0.702$
230	0.001	$0.702+\frac{-82.505}{\left(\frac{-0.758}{b(12.657+a+c)}+5.165\left(\frac{a}{\frac{-2.862}{b}+4.828+a}+ac\left(7.329\times10^{-4}\right)+b\right)+b\right)^2}$
250	0.001	$0.702+\frac{-82.566}{\left(\frac{-0.711}{b\left(\frac{21.092}{b}+a+c\right)}+5.167\left(\frac{a}{\frac{-2.831}{b}+4.813+a}+ac\left(7.328\times10^{-4}\right)+b\right)+b\right)^2}$
252	7.775×10^{-4}	$0.707+\frac{-87.316}{\left(a^2c\left(-1.405\times10^{-7}\right)+5.292\left(\frac{a}{\frac{a}{-33.302b+a}+b+a}+ac\left(7.823\times10^{-4}\right)+b\right)+b\right)^2}$
270	8.792×10^{-4}	$0.692+\frac{-189.836}{\left(b^3\left(4.522\times10^{-4}\right)\left(\frac{a}{-106.593+a+c}+91.310b\right)+8.152\left(ac\left(7.618\times10^{-4}\right)+\frac{a}{b+a+c}+b\right)+b\right)^2}$
277	0.001	$\frac{-83.997}{\left(5.202\left(\frac{a}{\frac{1}{-0.003a+ac\left(3.777\times10^{9}\right)+b}+b+a}+ac\left(7.335\times10^{-4}\right)+b\right)+a\left(1.002\times10^{-5}\right)+b\right)^2}+0.703$
277	0.001	$0.703+\frac{-86.242}{\left(5.283\left(ac\left(7.310\times10^{-4}\right)+\frac{a}{\frac{a}{4.781b^3+b+a}+b+a}+b\right)+a\left(6.976\times10^{-6}\right)+b\right)^2}$
279	6.384×10^{-4}	$\frac{-205.480}{\left(0.110b^2\left(0.004ac+\frac{a}{b+a+c}+b\right)+8.509\left(\frac{a}{3.319+a+c}+ac\left(7.138\times10^{-4}\right)+b\right)+b\right)^2}+0.680$
279	7.831×10^{-4}	$0.707+\frac{-87.117}{\left(5.285\left(\frac{a}{\frac{a}{\frac{1}{3.256\times10^{10}+b}+b+a}+b+a}+ac\left(7.825\times10^{-4}\right)+b\right)+a^2c\left(-1.398\times10^{-7}\right)+b\right)^2}$
283	9.969×10^{-4}	$\frac{-82.028}{\left(5.120\left(\frac{a}{-0.053ac+\frac{a}{-377.952+a+c}+b+a+c}+ac\left(7.498\times10^{-4}\right)+b\right)+ac\left(-2.850\times10^{-4}\right)+b\right)^2}+0.705$

continued on next page

Table S1 – continued from previous page (a: SeedInfected, b: R_0 and c: SeedInfectedDaily)

Complexity	1-R^2	Function
286	0.001	$\frac{-86.776}{\left(b^2 a\left(1.417\times10^{-6}\right)\left(-0.008ac+\frac{a}{-397.533+b+a}+b\right)+5.297\left(\frac{a}{1.976+a+c}+ac\left(7.443\times10^{-4}\right)+b\right)+b\right)^2}+0.703$
292	7.849×10^{-4}	$0.706+\frac{-87.975}{\left(a^2 c\left(-1.383\times10^{-7}\right)+5.319\left(\frac{a}{\frac{a}{-20.512b^3+b+a}+b+a}+ac\left(7.826\times10^{-4}\right)+b\right)+b\right)^2}$

Text S3: Symbolic Regression Analysis QALY

We focus on the surrogate modeling step of our approach with a dynamic transmission model to explore the cost-effectiveness of infant and adult VZV vaccination options. We analyzed the results from an economic evaluation with 185 inputs, 100 of which are correlated transmission rates using 1000 different configurations. First we performed a SR analysis with all 185 variables to model vaccination benefits, expressed in incremental QALYs. We observed that most surrogate models in the optimized high quality ensemble contain twelve parameters. In some cases, high dimensionality cause problems with the parameter selection in the sense that some less important variables remain captured in the surrogate models. Therefore, we performed a second SR analysis with these twelve variables to select the most important variables. The SR parameters are listed in Table 1 in the main text. All surrogate models obtained with SR are presented according to complexity and model error in Figure S1. We selected models in the knee of the Pareto front to obtain a high quality model ensemble with limited complexity. This ensemble is optimized using nonlinear techniques to end up with a predictive set, presented in Figure S1. Descriptive statistics for all surrogate models and for the optimized high quality model ensemble are given in Table S1. Table S3 illustrates surrogate model examples for the QALY.

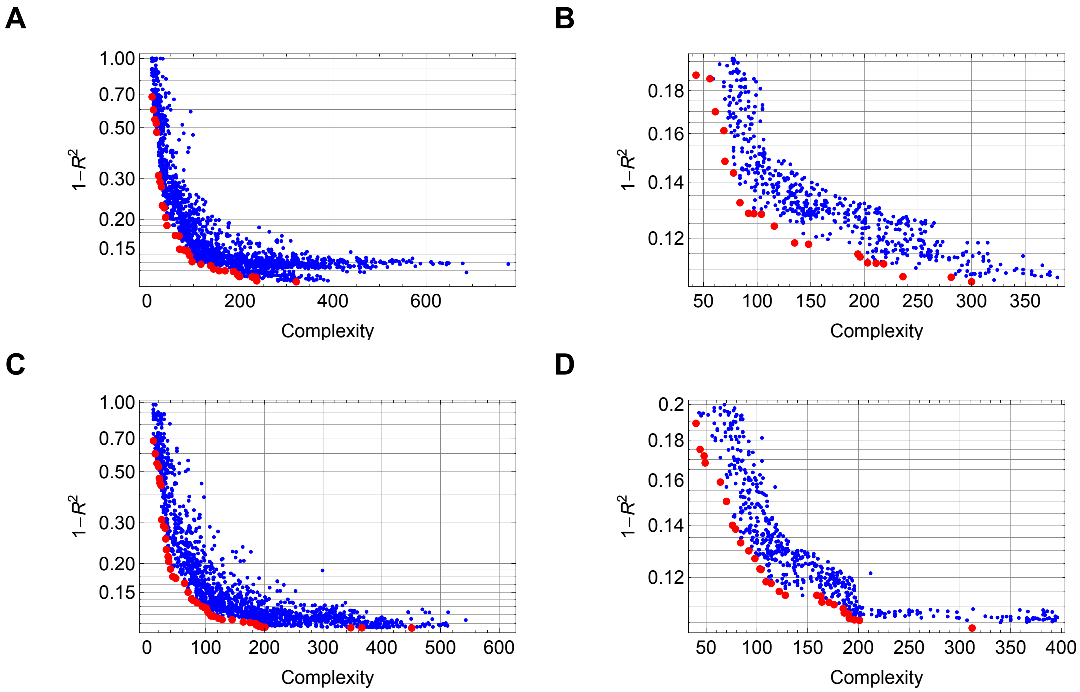

Figure S1. Model error (1-R^2) and complexity of the surrogate models for the QALY. The models indicated in red are on the Pareto front and the model error is presented in a log scale. A: SR analysis with all variables - B: SR analysis with all variables, optimized high quality ensemble - C: SR analysis with twelve selected variables - D: SR analysis with twelve selected variables, optimized high quality ensemble.
