# Supplementary material for: Active Learning to Understand Infectious Disease Models and Improve Policy Making
Source: PLoS Comput Biol. 2014 Apr 17;10(4):e1003563. doi: 10.1371/journal.pcbi.1003563 (PMC3990517; doi:10.1371/journal.pcbi.1003563)
Supplement: Text S4 — Response plot explorer. (PDF) [file pcbi.1003563.s005.pdf]

Text S4: Response Plot Explorer

In order to improve confidence in model-based conclusions, it is necessary to gain a thorough understanding of the system and assess how model assumptions and parameters alter the results. Surrogate models are very useful for this purpose since they can be readily explored. We used Pareto-aware symbolic regression to analyze input-response data to obtain algebraic expressions. These can easily be used in a visualization tool to explore the response surfaces from multiple parameters. Parameter must be chosen and the predicted response plots are shown for every parameter with all others fixed. Figure S1 and S2 presents screen-shots of a response exploration tool we installed at *www.idm.uantwerpen.be* with surrogate model for the cumulative clinical attack rate from FluTE. Surrogate modeling is relevant for many public health problems. Figure S3 shows an analogous tool to estimate the quality adjusted life year gain of varicella-zoster virus vaccination.

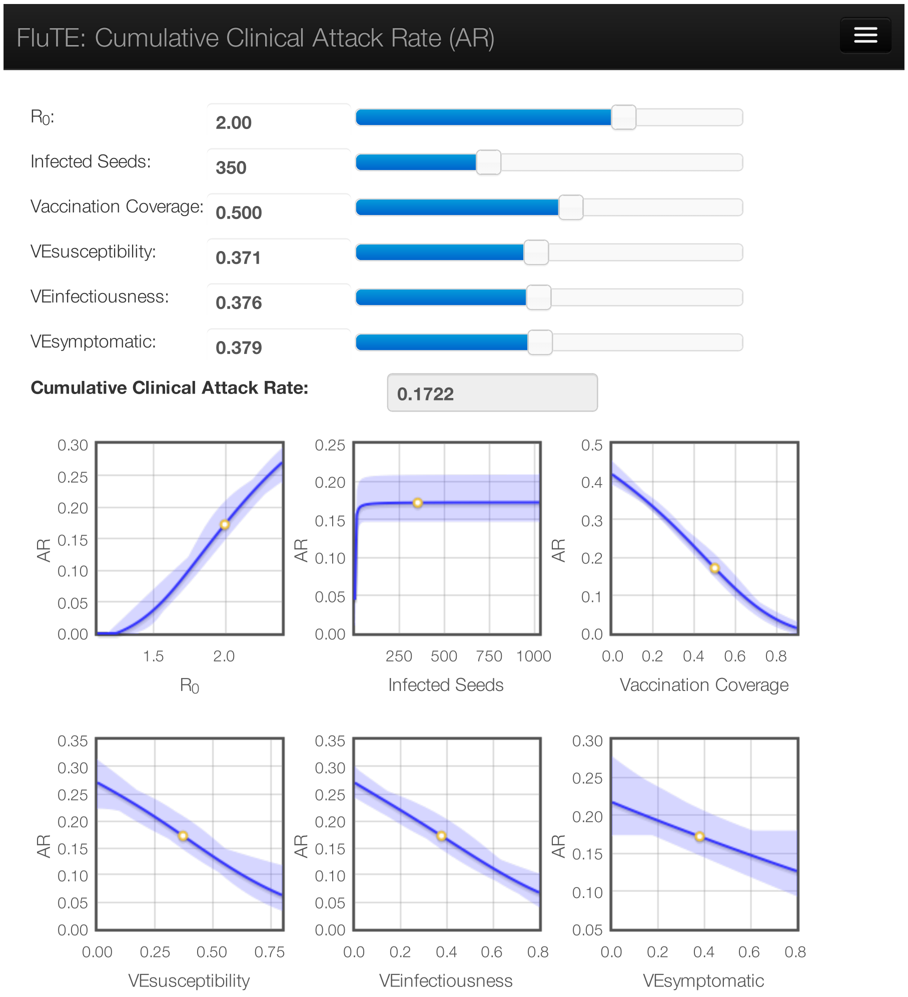

Figure S1. Response plot explorer for the cumulative clinical attack rate. The shaded area presents the model ensemble divergence, a representation of the prediction uncertainty. An interactive version is available at *www.idm.uantwerpen.be*.

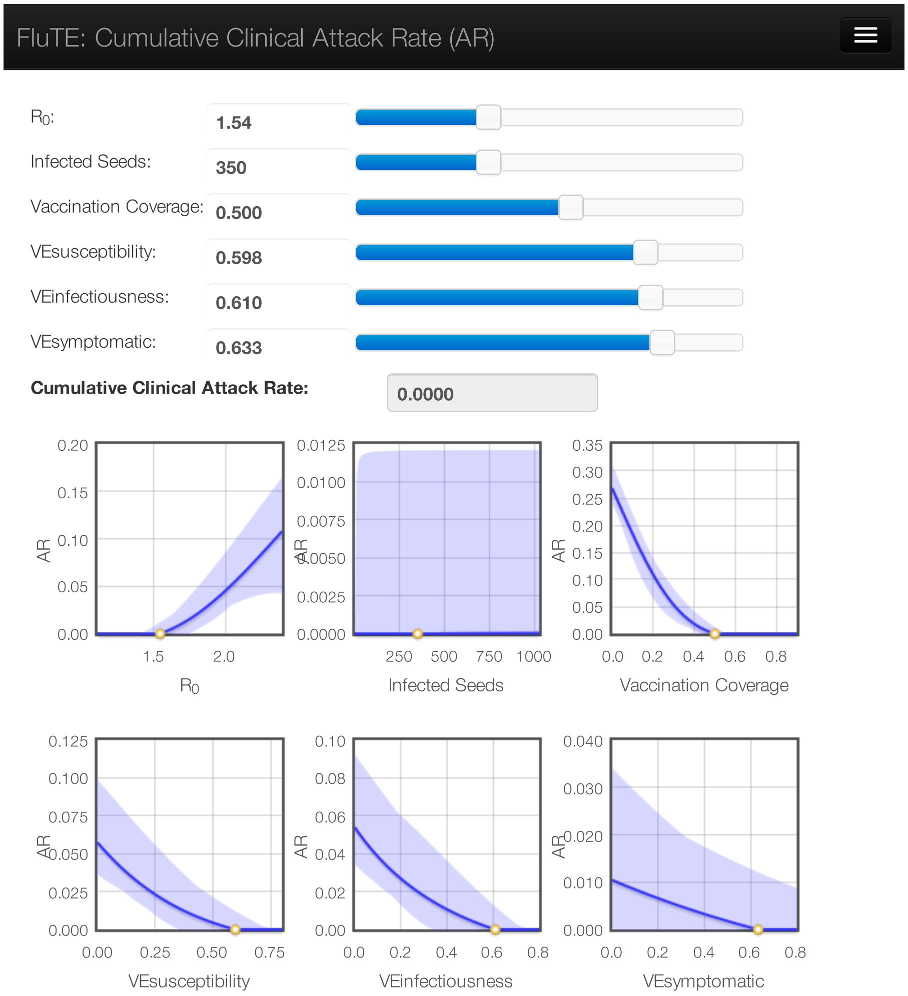

Figure S2. Observing herd immunity with the response plot explorer for the cumulative clinical attack rate. Limited R_0 and high vaccine efficacies result in total protection of the population (AR=0) with only 50% vaccination coverage. This is known as herd immunity.

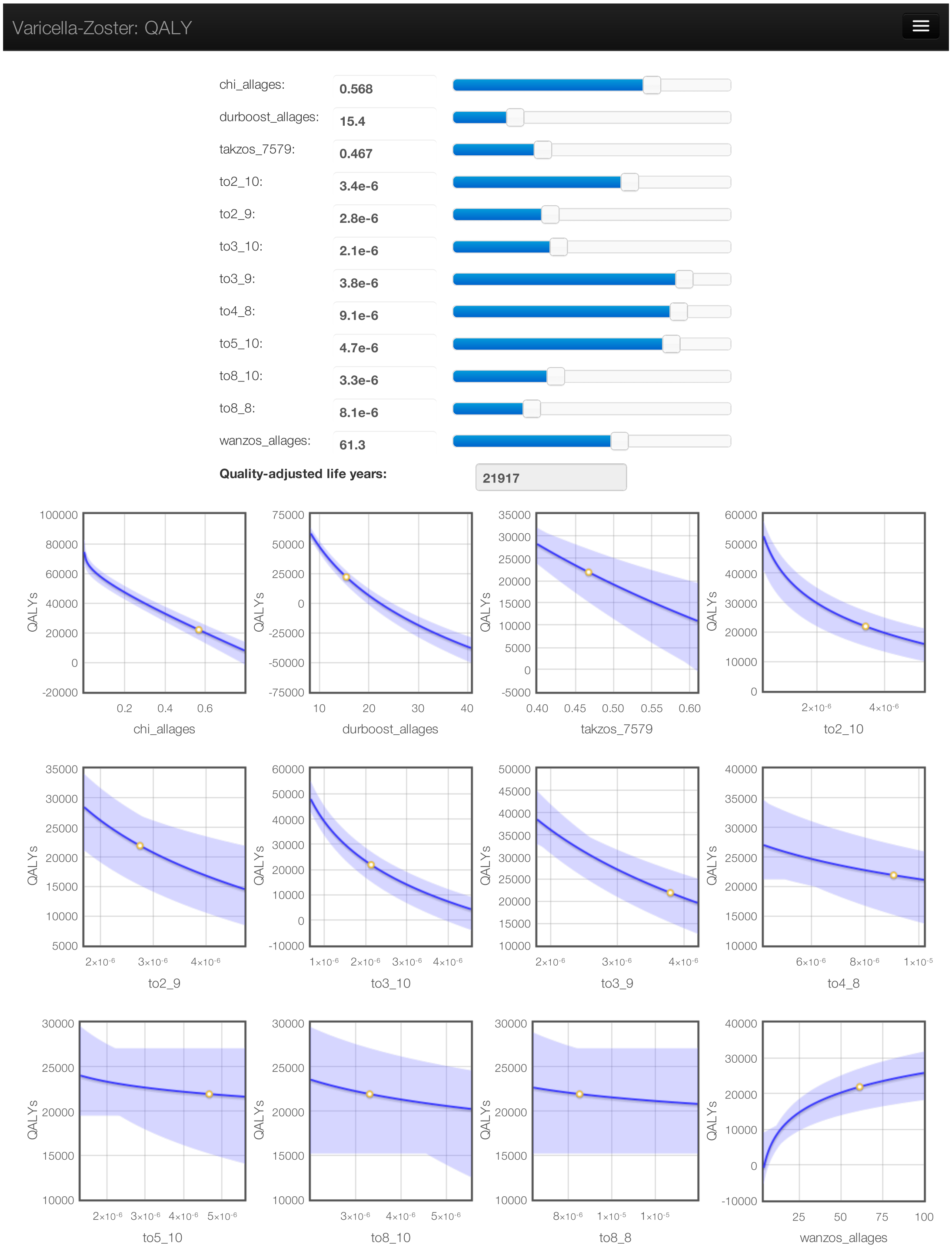

Figure S3. Response plot explorer for the quality adjusted life year gain of varicella-zoster vaccination. An interactive version is available at *www.idm.uantwerpen.be*.
